# TCP BBR-n: Increased throughput for wireless-AC networks

**DOI:** 10.1371/journal.pone.0295576

**Published:** 2023-12-11

**Authors:** Muhammad Ahsan, Sajid S. Muhammad

**Affiliations:** Department of Electrical Engineering, National University of Computer & Emerging Sciences, Lahore, Punjab, Pakistan; Seoul National University of Science & Technology, REPUBLIC OF KOREA

## Abstract

Google proposed a new TCP congestion control algorithm (CCA), Bottleneck Bandwidth and Round-trip propagation time (BBR) which has opened up new dimensions in congestion control. BBR tries to operate near Kleinrock’s operating point to avoid excessive queue formation at the bottleneck and to use the link bandwidth optimally. BBR creates a model of the network path by measuring the bottleneck bandwidth and minimum round-trip time (RTT) to maximize the delivery rate and minimize latency. BBR v2 is an updated version of BBR which addresses many shortcomings of the original BBR (BBR v1) such as interprotocol fairness, RTT fairness, and excessive retransmissions. However, BBR v2 has certain limitations in its operation in IEEE 802.11ac (Wi-Fi 5) networks. The default BBR v2 limits the throughput of Wi-Fi 5 and an increased latency has been observed. This is because the Wi-Fi 5 frame aggregation logic is underutilized and fewer frames are being sent to the Wi-Fi 5 interface. In this paper, we have proposed BBR-n (BBR new) which provides better throughput than the generic BBR v2 in the Wi-Fi 5 networks. Real-time experiments were performed over a physical testbed using Flent to confirm that BBR-n achieves over double throughput as compared to generic BBR v2 and reduced latency in networks as compared to pure loss-based variants such as Cubic and Reno.

## 1 Introduction

**TCP** (Transmission Control Protocol) and its associated congestion control (CC) algorithms have been around since 1980. We have Tahoe, Reno, Vegas, Compound TCP, Data Center TCP, Cubic, Veno, and many others. Cubic is the current most deployed congestion control algorithm in Microsoft Windows 10 and Linux kernel. All these mechanisms were mostly based on detecting congestion with packet loss and duplicate acknowledgments (ACKs). These congestion control techniques were good in the early days of the internet when bandwidth was less, and delays were relatively high as compared to today’s fast internet. But with modern internet with high-capacity links having higher bandwidth and relatively lower latency, these loss-based techniques have proven to reduce performance. Hence, there is a need to fine-tune the congestion control algorithms (CCAs) and propose some new ones that can better utilize the huge bandwidth that today’s broadband networks present.

TCP Cubic was then proposed, which proved beneficial due to its more aggressive strategy of engaging bandwidth but had issues such as bufferbloat [1]. These CC algorithms over time have proven less beneficial in terms of handling the higher bandwidth optimally and reducing the Round-trip times (RTTs). Being focused on viewing congestion because of packet loss only has resulted in issues like shallow buffers (due to commodity switches), bufferbloat phenomena at the network edge, and various other factors due to the dynamic nature of internet traffic.

Although Transmission Control Protocols’[2] congestion control (CC) algorithms are evolving, but not at the pace at which the network infrastructure and the associated bandwidth are rising. The CC algorithms are being proposed time and again to unleash the full potential of available bandwidth and to keep the channel utilization near its maximum [3]. In 2016, Google proposed an algorithm named Bottleneck Bandwidth and Round-trip-time (BBR) and it claims that its algorithm provides better throughput and lower latency [4–6]. Google is right now using it in its YouTube servers and B4 backbone [7]. It attempts to work near Kleinrock’s operating point [8] and as a result gives better throughput.

**Fig 1:**
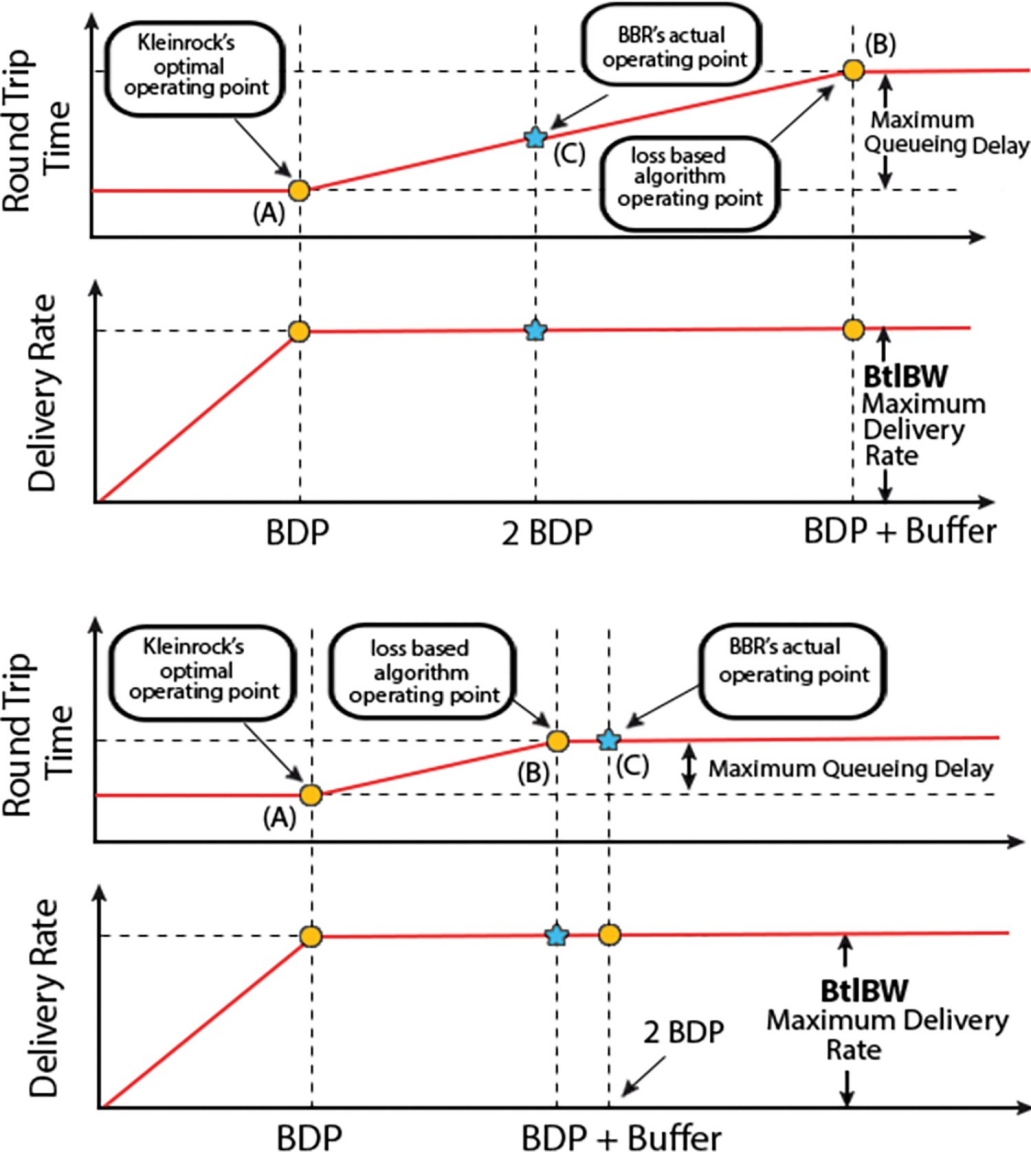
(a). Buffer size > 1 BDP. (b). Buffer size < 1 BDP.

**“Fig 1A”** depicts the relation between delivery rate and Round-trip time (RTT), which is the Bandwidth Delay Product (BDP). We see that the delivery rate increases linearly to point A (Kleinrock’s optimal operating point) [9] and the data in flight increases. During this period of linear increase, the RTT remains almost constant. From point A onwards the throughput remains the same, but RTT starts to rise. From this time onwards the buffer is being filled and a queue is being formed. At point B, packet loss occurs as the buffers are full and RTT is no longer increasing.

The pure loss-based algorithms try to operate at point B, whereas BBR v2, which uses a hybrid approach tries to operate at Kleinrock’s operating point by estimating the BDP dynamically. It tries to keep the congestion window (cwnd) to 2 * BDP so its actual operating point becomes point C as shown in “**Fig 1A”**. The buffer size here is larger than 1 BDP. This is a typical case of deep buffers [10]. But when the buffer size is less than 1 BDP the operating point shifts to the right of B, as shown in “**Fig 1B”** which results in packet loss.

As we can see this is a problem, as BBR v2 does not reduce the cwnd based on packet loss only, but it actively and dynamically calculates the sending rate by probing the available Bottleneck Bandwidth (BtlBw) and the Round-Trip-Propagation delay (RTprop) in real-time with the help of acknowledgments (ACKs). This model-based [11] approach of BBR v2 results in a good performance for wired networks, but we will prove that in the case of Wi-Fi 5 networks the throughput is reduced. Here it is pertinent to mention that BBR v2 does use packet loss and DCTCP-inspired Explicit Congestion Notification (ECN) signals (if they occur) [12]. Although BBR v2 can use ECN or loss signals explicitly, it does not require either; it can bound its in-flight data based on its estimate of the BDP. We decided to name this default BBR v2 as *generic BBR* (BBRgen). So, BBRgen will refer to BBR v2 throughout this paper.

BBR v2 has been designed using wired bottleneck models. It does not work well with Wi-Fi technologies such as 802.11n/ac/ax, as it breaks the frame aggregation logic of the standard model. Although the mechanism of packing together several frames and sending them in one transmission unit started from 802.11n onwards, its real advantage is achieved in new technology such as 802.11ac. The Wireless AC uses MU-MIMO (Multi-user, multiple-input, multiple-output) which enhances the Wi-Fi experience by providing better throughputs. Moreover, Wireless AC adapters are now comparatively cheaper than 802.11ax, which is the main reason why we are focusing on Wireless AC for our paper. IEEE 802.11ac expands the maximum aggregation size of 65.5 Kbytes in 802.11n to 1 Mbyte in Wireless AC [13]. The default BBR v2, as we will discuss in detail in this paper is compromising the frame assembling logic of Wireless AC and does not assemble enough frames at a swifter pace to make a bigger quantum of data that the Wireless AC is capable of handling. This results in an inefficient performance of Wi-Fi 5 when BBR v2 is used as a congestion control algorithm over it. Our proposed algorithms work with both 802.11n and 802.11ac, but for the sake of brevity tests from 802.11n are included in our data repository [14] and we focus on tests with Wireless AC in this paper. It will work on 802.11ax as well as this technology using the aggregation mechanism, but in this paper, we are focused on tests on Wireless AC only.

For optimum frame assembling in Wireless AC when using TCP BBR v2, the initial startup phase of the generic BBR needs revision. The proper selection of the size of the data burst, how much data to enqueue, and when to send this data are the additional critical steps. The novelty of this paper is the proposed BBR-n that incorporates all these important factors and provides the enhancement in throughput for Wireless AC and lower latency as compared to BBR gen.

The contributions of this paper are the following: (i) We prove that degradation in throughput arises when TCP BBR v2 is used with a Wi-Fi 5 network. Considering the topology when the client is connected via the Wi-Fi 5 and the server is wired. (ii) We develop a revised version of BBR v2 which we call BBR-n (BBR new), it consists of four components. (a) An analytically derived startup gain value for BBR v2 flows in their startup phase to get a smoothly increasing pacing rate that doubles every RTT, maintaining fairness with any un-paced Cubic and Reno flows going alongside. This new startup gain value which allows an optimum increasing pacing rate has been verified via discrete simulator results in Section 3.2.1 (b) Our TCP small queues (TSQ) implementation for Linux kernel for relaxing the hard-coded low TSQ limit of 1 ms in Linux kernels 5.x.x. This allows the TSQ to enqueue a much larger aggregate of data that will allow the Wi-Fi 5 to unleash its maximum throughput. (c) We propose *Algorithm 1a*. to modify the probing for bandwidth (ProbeBW) phase of BBR v2 so that the bottleneck when using aggregation policies can take full advantage of it to get higher throughput while maintaining minimum queues. (d) The crucial role of Linux kernel’s TSQ and transmission/generic segmentation offload mechanisms (TSO/GSO) affecting the Wi-Fi 5 frame aggregation are also unleashed via our proposed *Algorithm 1b*. in which a larger quantum of TCP maximum segment size (MSS) has been proposed to fully utilize the relaxed TSQ limit. (iii) BBR-n has been tested on our physical testbed and increased throughput with relatively reduced latency as compared to loss-based CCAs has been achieved.

The remaining part of our paper is sectioned as follows: Section 2 sheds light on the related works done in this context. Section 3 provides insight into the TCP architecture of the Linux system, details on default BBR v2, and the proposed BBR-n. Section 4 provides the details on the physical testbed deployed to generate the results which are discussed in Section 5. Finally, we have Section 6 which provides the conclusion of this paper along with the future directions.

## 2 Related works

Development work on TCP BBR v2 is actively in process in the internet industry. Google is successfully using it in its internal WANs for its YouTube servers and B4 backbone with major gains achieved in throughput. More work is still in progress for its implementation on endpoints, especially in a wireless scenario. Performance evaluation for BBR v1 with popular loss-based congestion control algorithms on a Long-Term Evolution (LTE) uplink and cellular networks have been performed in [15–17]. With multiple flows present along BBR v1, the issue of fairness resulted in a queue buildup, and packet loss was experienced. In several network scenarios, BBR v1 does provide better throughput as compared to loss-based congestion control algorithms such as Reno and Cubic, but in LTE uplink and cellular networks, this was not the case, and multiple flow traffic caused excessive queue buildup at the bottleneck.

Yeong-Jun Song et al. [18] propose BBR v1 congestion window scaling (BBR-CWS) which mitigates BBR v1 limitations when Cubic or Reno flows are going along in an emulated wired network scenario. It provides a scaling technique so that BBR v1 and loss-based CCAs can coexist and claim to reduce packet re-transmissions. A variant of BBR v1, known as BBR Advanced (BBR-A) tackles the fairness and re-transmission challenge of BBR v1 and provides better results with experiments done using the Mininet emulator [19]. Modest-BBR is another flavor of BBR v1 that focuses on reducing its pace and bridging the fairness gap between BBR v1 and Cubic [20]. BBR-CWS, BBR-A, and Modest-BBR bring improvement in BBR v1 working by improving fairness and reducing transmissions, but the work done is on a wired scenario only.

BBR v2, whose alpha code has been released and Google has deployed it for all of its internal production TCP traffic [21]. It tackles the issues of fairness and re-transmission in BBR v1. For external TCP traffic Google is still using BBR v1 and slowly making the transition to BBR v2. Research work on BBR v2 is going on and Wansu Pan et al. [22] proposed a flow-aware ECN mechanism to improve the RTT fairness issue that may arise when multiple BBR v2 flows enter the same link at different times. This work was done using network Simulator 3 (NS3) and no physical testbed was involved. BBR v1 and v2 were evaluated by Zhang [23] and it showed that BBR v2 performed better than BBR v1 in terms of RTT fairness and an improved coexistence with Cubic and Reno flows. NS3 was used to get the simulation results in a wired network topology here as well. Active research on BBR v2 is continuing and researchers are using simulation environments and mostly wired topology. The reason behind this limitation is that the recipe to build the Linux kernel with TCP BBR v2 support at this early stage is targeted for bare metal or Google Compute Engine (GCE) test machines. Also, the work done on BBR v2 is mostly on a wired simulated networks with very less work done on BBR v2 in a wireless environment, especially the Wi-Fi 5. Our work involves a physical testbed with Linux kernel 5.15.72 built with BBR v2 as a loadable module for real-time testing with BBR v1, Cubic, Reno, DCTCP, Veno, etc. in a wireless scenario, where the client is connected via Wi-Fi 5 and the server is a wired Linux machine.

In Wireless LANs (WLANs) especially the most common Wi-Fi 5 standard, frame aggregation mechanisms that were introduced with it have been probed via simulations and analytical methods [24,25]. Less work has been done on improving TCP BBR v2 performance over Wireless LANs as compared to fixed LANs. Another BBR variant, BBRp [26] tries to tackle BBR issues by taking into account the TCP pacing gain and uses an algorithm in which the variable *bbrp_pace* was introduced to control the pacing gain. Our probing on this issue led to some interesting findings, the *bbrp_pace* variable proposed was producing a pacing gain value of 1.5, and the drain gain was left unchanged in the algorithm as 0.75. This can have an impact on cross traffic and can result in reduced throughput, as the corresponding drain gain was not revised in this case. Moreover, BBRp is not responding to any changes in TSQ due to not enough segments being generated by the TSO engine of generic BBR [27].

The author of TCP BBR, Neal Cardwell proposed some fixes earlier than BBRp to improve the performance of TCP BBRv2 in WLANs in a group named BBR Development [28], but his patches were focused on the scenario when the client is on a fixed network and server is connected via a Wi-Fi link. Our results show that the pacing gain and an adaptive drain gain in generic BBR are too conservative in the case of Wi-Fi 5 in which larger chunks are needed to build bigger frames to take advantage of the frame aggregation logic inherently present in this technology. We resolved this issue by providing a solution that works in the opposite scenario in which the client endpoint is using a Wi-Fi 5 connectivity and the remote server is wired.

During our research on increasing the throughput of Wi-Fi 5 networks using BBR v2, we found via rigorous experiments on our physical testbed that if the pacing gain changes, there needs to be a corresponding change in the drain gain as well. This is to balance the average pacing gain. This is missing in [29] and results show that this leads to a decreased throughput for Wi-Fi 5 networks. The balance between choosing the pacing gain and drain gain is very critical as the larger pacing gain can cause large queues (if the bottleneck link is full) and these larger queuing delays and potentially larger losses impact cross-traffic. The works done till now have been summarized in **“Table 1”** for the reader’s glance.

**Table 1:** Related work in a nutshell.

Related Work	Re-transmissions Or Fairness	Wi-Fi	Ref.
BBR-CWS	Improved fairness and less re-transmission with cubic flows.	None	[18]
BBR-A	Improved fairness and less re-transmission with cubic flows.	None	[19]
Modest-BBR	Better performance with Cubic flows.	None	[20]
BBRv1	Fairness issues with Cubic and Reno flow going along.	None	[15–17]
BBRv2	Tackles interprotocol fairness and retransmission issues of BBR v1.	In the Wi-Fi case, it assumes that the server is on a Wi-Fi link.	[28]
BBRv2+	Claims to resolve fairness issues in BBR v2 by flow-aware ECN.	None	[22]
BBRp	Imbalance between pacing gain and drain gain.	Fails to respond to TSQ due to less TSO budget.	[26]
BBR-n	Fairness validation via RRUL test in both uplink and downlink paths.	Provides Increased throughput for IEEE 802.11ac networks with reduced latency as compared to BBRgen.	[14]

Our work solves the limitations of generic BBR of reduced throughput in Wi-Fi 5 by deriving a revised startup gain value that BBR-n uses initially to rapidly probe the highest available bandwidth in the smoothest possible fashion. Our proposed *Algorithm 1a*. provides a better strategy for the pacing gain & *Algorithm 1b*. helps in making an optimum MSS budget that suits the Wi-Fi 5 frame aggregation logic together with the relaxed buffer size of 4 ms via the TSQ patch.

## 3 BBR v2 and BBR-n

Linux TCP stack is highly enriched with many features. It is the main reason it’s being used in millions and billions of devices around the world. We focus here on the most important of its features, its congestion control, segmentation, and pacing mechanisms.

**Fig 2:**
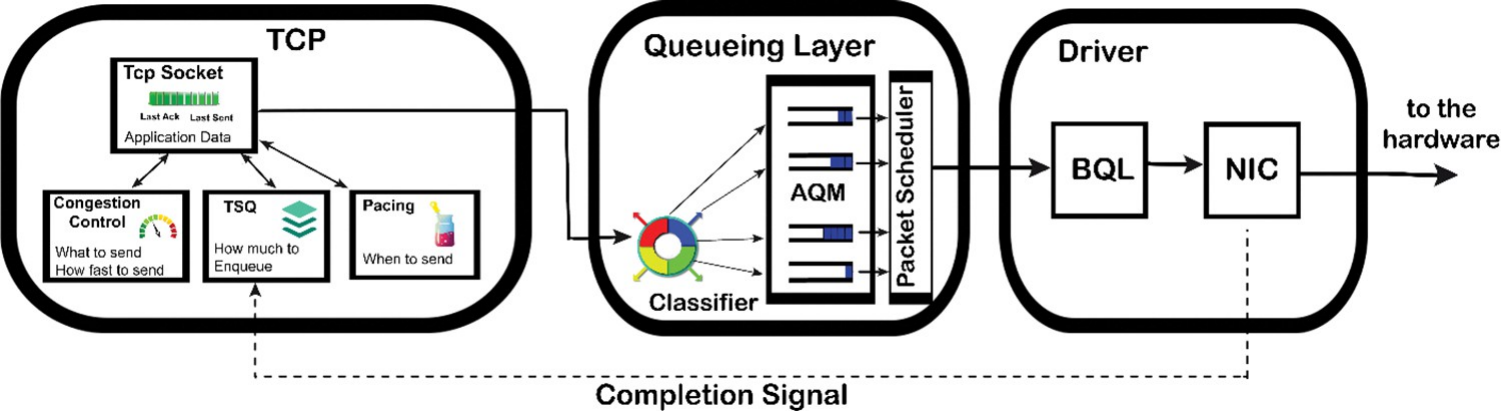
TCP in Linux kernel.

BBR v2 alpha has been rebased to Linux kernel 5.10 [30] and work on it is in the process to streamline it for all types of networking scenarios. Before diving deep into BBR v2 architecture, it is important to know basic Linux TCP architecture. BBR v2, which is a model-based congestion control algorithm is very smart and uses its own segmentation and pacing logic, which is different from the one Linux TCP uses. All loss-based congestion control algorithms such as Cubic and Reno also follow the Linux TCP. TCP in the Linux kernel as shown in “**Fig 2”** [26] has various sub-modules to take care of the segments generated. On the top, we have TCP sockets with Congestion Control, TCP small queues (TSQ), and the pacing rate sub-modules linked with it in a bi-directional manner. At the queuing layer, we have FQ (Fair Queue) and FQ-Codel (Fair Queuing with Controlled Delay), which is a hybrid packet scheduler and active queue management (AQM) [31] algorithm to mitigate bufferbloat and reduce the latency [32]. Next is the Ethernet/Wi-Fi driver module which physically forwards the packets to the network interface controller (NIC) hardware logic to make data link layer frames and send them over the physical transmission link as raw bits.

Here it is pertinent to mention other approaches like in [31] where CoDel has been proposed to tackle the issue of persistently full buffers in the internet. It uses packet-sojourn time through the queue instead of the queue size to keep track of the filled queues. To reduce internet congestion using feedback mechanisms, Proportional Integral Controller Enhanced (PIE) AQM has been around as well. PIE combined the benefits of both Random Early Detect (RED) and CoDel [33]. It is a latency-based design to control the issue of bufferbloat. PIE drops packets based on the current queuing delay and the delay moving trend. It has a parameters self-tuning feature to optimize system performance. It is worth mentioning that only linear approximations with constant delays have been mostly used to propose various AQMs such as RED, PIE, and those proposed in [34,35]. H. Mounier et al. [36] propose an AQM control-theoretic method in which the constraint of assuming a constant delay, i.e. the round trip time has been lifted and a non-linear model with a variable delay has been introduced. It discusses the control-theoretic tools to handle nonlinear modeling by using open-loop control and closing the loop by Model-Free Control (MFC).

Most recently, we have Common Applications Kept Enhanced (CAKE) [37] AQM introduced by the bufferbloat project which is best suited for home gateways. It combines traffic shaping and AQM in a single algorithm. Improving on FQ_CoDel, CAKE reduces CPU utilization by reducing hash collisions between flows. Although TCP with its CCA helps mitigate the congestion at the transport layer, we need to have a suitable AQM deployed at the network layer to avoid the buffer overflow in the routers. It is indeed this TCP-AQM combination that can provide the most effective congestion control. More research work needs to be done in this case in which an ideal combination of TCP and AQM should be identified that can mitigate the congestion more effectively than TCP CCA alone. Since the internet is a heterogeneous network a “one size fits all” solution does not exist. Researchers may suggest an optimum TCP-AQM combination for a particular network scenario e.g. in a typical home wireless bottleneck.

### 3.1 BBR v2

**Fig 3:**
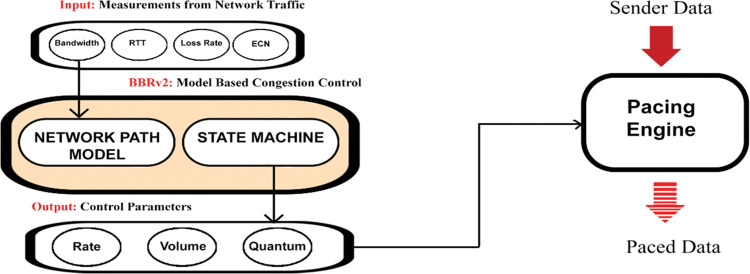
BBR v2 architecture diagram.

**“Fig 3”** shows the working of TCP BBR v2 from a higher-level perspective. It can be seen that BBR v2 is modeled around the Bottleneck Bandwidth and the Round-trip time, to maximize the former and minimize the latter. It also uses loss and DCTCP-inspired ECN, if they occur. For that purpose, the BBR v2 algorithm uses the results of BtlBw (throughput), RTprop (delay), ECN, and any packet loss estimates that are fed as input to the BBR v2 state machine [38].

**Fig 4:**
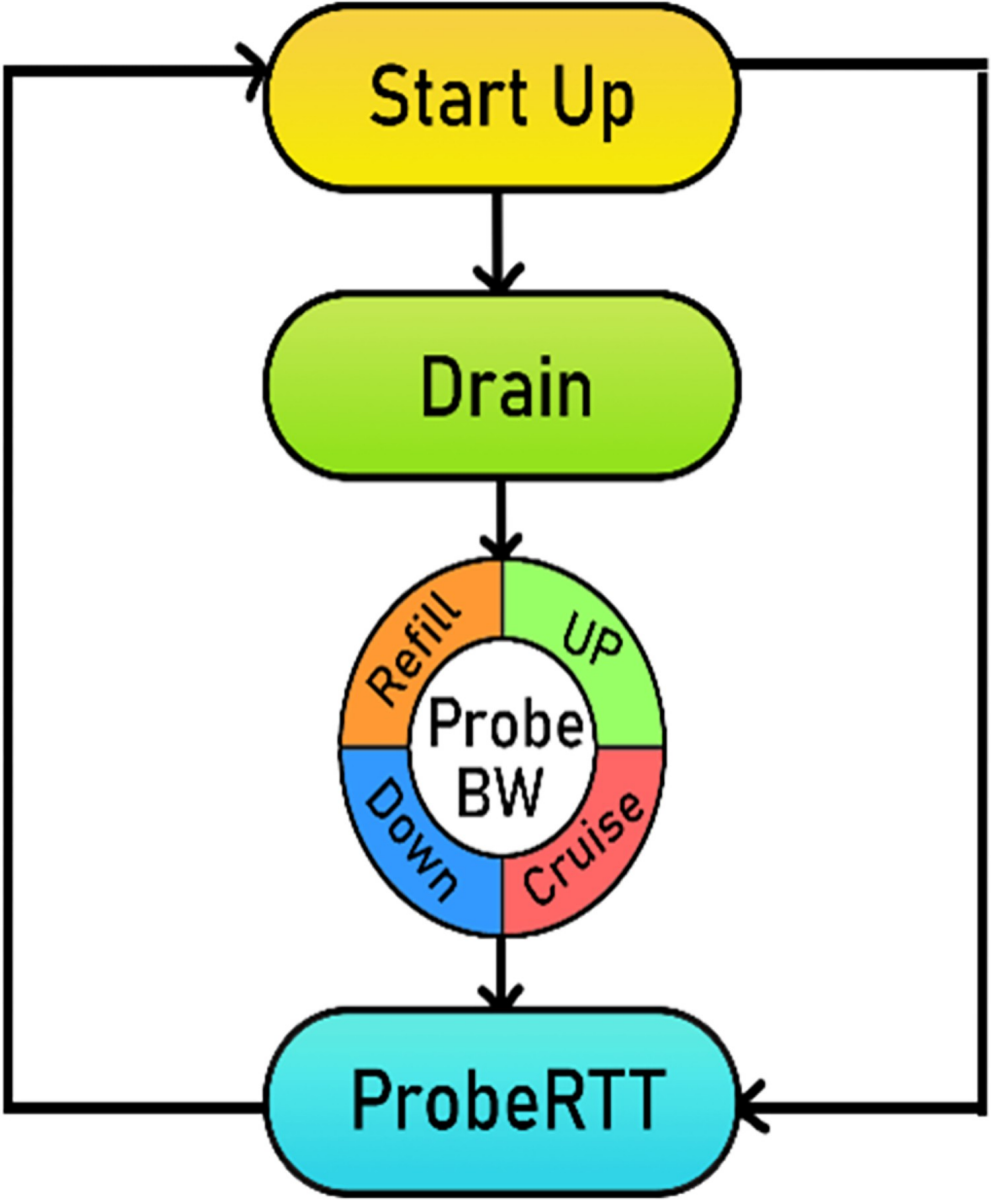
BBR v2 state machine.

As a result, the rate (pacing rate which controls inter-packet spacing), quantum (which is the maximum size of data aggregate), and volume (which is the cwnd size estimate) are set. These are the inputs to the sending engine to pace the incoming data and keep it near the BDP so that the pipe remains optimally filled with data and consequently higher utilization is achieved. The BBR v2 congestion control algorithm working is based on the estimation of the Bottleneck bandwidth, Round-trip time, packet loss, and ECN in real time. These are the basic input parameters to the BBR v2 probing state machine, whose four main states (Startup, Drain, ProbeBW, ProbeRTT) are shown in the state diagram in “**Fig 4”.** The ProbeBW cycles through the four ProbeBW sub-states – DOWN, CRUISE, REFILL, and UP. In the steady state, a BBR v2 flow only uses ProbeBW and ProbeRTT. A long-lived flow spends most of its time in the ProbeBW phase looking for available bandwidth to keep the pipe full and minimize the queue.

In the ProbeBW: DOWN phase BBR v2 shifts down its packet sending rate into the network to make sure the data in flight is reduced. It does so by lowering the pacing gain value to 0.9, sending at 90% of the BBR bandwidth (BBR.bw). The flow exists in this phase to enter CRUISE, if it sees that there is free headroom or it estimates that it has drained any queues at the bottleneck.

In the ProbeBW: CRUISE phase the BBR v2 flows tries to send at the same rate the network is delivering data. It does so by switching to the pacing gain value of 1.0, sending at 100% of BBR bandwidth (BBR.bw). It then holds this state adaptively and responds to any packet loss signals.

The goal of the ProbeBW: REFILL phase is to refill the pipe, to try to fully utilize the network bottleneck, and to avoid any queue pressure.

In the ProbeBW: UP phase if the packet loss exceeds the 2% threshold value or the filtered ECN rate is more than the 50% threshold value, BBR v2 will estimate the max safe inflight volume by setting *inflight_hi* [11] and leave the unused headroom. To achieve this it uses a pacing gain value of 1.25, sending faster than the current estimated bandwidth. To ensure fairness with pure loss-based CCAs, the congestion window size in the ProbeRTT phase of BBR v2 has been reduced to half of the inflight.

The basic role of the BBR v2 state machine is to increase/decrease the inflight data around the target inflight. Where target inflight equals the estimated bandwidth-delay product (BBR.BtlBw * BBR.RTprop). In the Startup state, BBR v2 flow rises exponentially like Cubic, doubling the cwnd after every RTT until it reaches the bottleneck bandwidth. In the Drain state which is one RTT delayed, the queue has already been formed and it tries to drain the queue by decreasing the pacing gain. It then enters the ProbeBW phase and starts probing for more available bandwidth, for this probing cycle BBR v2 has four sub-states.

The BBR v2 has its pacing gain [11] paced at four cyclic values, BBR v2 keeps sampling the bandwidth and getting values for new BtlBW from the windowed maximum recent bandwidth sample – obtained using the BBR v2 delivery rate sampling algorithm [39]. We will soon see in the next sections that the pacing gain values in the ProbeBW phase are very conservative and do not suit for Wi-Fi 5 scenario in which frame aggregation [40] is used to deliver higher throughput. These cyclic values reduce the throughput by breaking the frame aggregation logic. In the last phase of the BBR v2 state machine, it enters the ProbeRTT phase if the value of RTT is too old, stops probing for more bandwidth, and tries to get a new estimate of the RTprop which is the valid for next five seconds.

### 3.2 BBR-n startup gain

TCP small queues is a relatively newer algorithm introduced by Google that provides the necessary flow control mechanism to the flows going on the sending host. To do so each flow enqueues a limited amount of packets in its buffers to avoid the Bufferboat [1] and overwhelming the sender node queues. It only gets the new packet once the NIC has dispatched the packet. Now in the current Linux TCP stack, TSQ is allowed to enqueue as much data that can be transferred in an interval of 1 ms for any flow’s current sending rate. This standard value of 1 ms although good for a wired network scenario is too strict when it comes to Wi-Fi 5 networks where frame aggregation is hampered by such a limit. We need more frames in a Wi-Fi 5 scenario so that frame aggregation is done optimally.

Along with TSQ, the BBR v2 startup gain, which is a dynamic gain factor used to scale and produce the TCP pacing rate is a crucial factor. It is the current pacing rate for a particular flow that controls the inter-packet spacing. The current value in the startup phase for this gain as proposed by Neal Cardwell [4] in his latest September 2022 draft on BBRv2 is 4*ln(2) ~= 2.773. Although this value is reasonable for fixed WANs, we found it was not optimum for Wi-Fi 5, so a revised startup gain is derived mathematically in section 3.2.1, and it is validated by simulation in section 3.2.2.

#### 3.2.1 Startup gain analytic derivation

Pacing is a mechanism used in Linux TCP to pace the packets down the stack layers. TSO on one end ensures the burst size and TSQ on the other end takes care of how many packets can be enqueued. The role of pacing is critical in controlling the internal rate of moving packets. This helps in mitigating the formation of bursts leading to the bufferbloat phenomenon. All the congestion control algorithms in Linux follow the Linux default pacing algorithm but BBR has its own. Linux as a default works at 200% in the slow start phase and at 120% in the congestion avoidance phase. BBR uses its own hardcoded rate. Since BBR is a model-based algorithm its pacing mechanism is different from loss-based algorithms. BBR pacing has BBR startup gain to control the startup phase of the flow ensuring that it produces a smoothly increasing pacing rate that would be sending the same number of packets per RTT as a Cubic or Reno flow would do so. In the second phase of probing for bandwidth, BBR has its unique way of using certain cyclic values for its gain and drain queues. BBR spends most of its time probing for the bandwidth phase and ensuring the pacing rate is optimum to provide a realistic delivery rate that matches the currently available bandwidth at that time. In this way, not only does it prevent the bloating of the queues but also provides optimum bandwidth for a flow.

In the startup stage of BBR v2 flow, the pacing gain has the following property:

Its rate of change of pacing rate is equal to the current pacing rate. So, let us assume ‘x’ is the time and keeping RTT constant, the BDP evolves as the following function F_1_(x):

$$F_{1}(x)=2^{x}$$ (1)

The pacing rate evolves as the function of time:

$$F_{2}(x)=2^{x}$$ (2)

Let g(x) be the derivative of F_1_(x):

g(x)=F1'(x)=ln2*2x (3)

The derivative of F_1_’ (x)= is the rate, so g(x) is the pacing rate.

Let ‘G’ be the pacing gain, based on BBR v2 architecture:

$$g(x+1)=G\;*\;g(x)=G\;*\;ln2\;*\;2^{x}=F_{2}(x+1)=2\;*\;2^{x}$$ (4)

So,

G * ln2 * 2^x^ = 2 * 2^x^

G=2/ln2=2.885 (5)

This value of G is found to be effective in Wi-Fi 5 and is incorporated in our patch for BBR-n.

#### 3.2.2 Startup gain validation via discrete simulator

**Fig 5:**
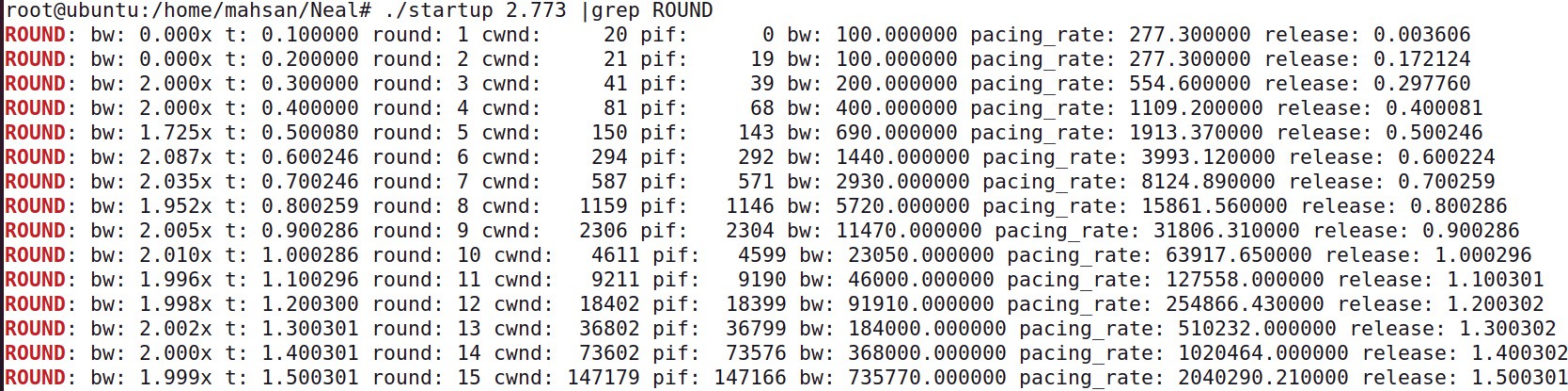
Gain = 2.773, discrete simulator.

**Fig 6:**
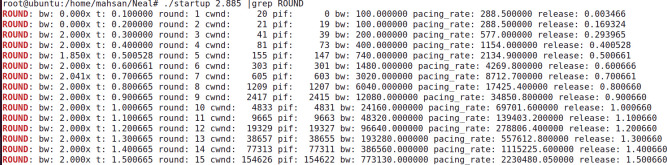
Gain = 2.885, discrete simulator.

The analytic derivation of the BBR v2 startup gain is validated via a discrete simulator, whose code is also shared here [41]. As seen from “**Figs 5** and **6”**, which are produced for Gain values 2.773 and 2.885 respectively. We see that the bandwidth (**bw**) is doubled in rounds 10-15. It smoothly doubled with a Gain value of 2.8885. Due to space limitations, results for various other gain values are shared at cloud data sources [14].

These results prove our assertion that a value of 2 / ln (2) is optimum for BBR’s initial start-up phase in doubling the bandwidth more smoothly. As suggested by Neal Cardwell [11], one other purpose of this Gain value is to make the pacing rate rise smoothly and double with each RTT, sending the same number of packets per RTT as they are sent in by TCP Reno and Cubic. This ensures fairness among the flows [18,42].

### 3.3 BBR-n (pacing gain and quantum selection algorithm)

We propose here BBR-n, which is based on BBR v2 and tackles this low throughput issue more specifically in the following ways. Along with the revised BBR v2 startup gain derived analytically and proved by the discrete simulator, we introduce *Algorithm 1a*. in which we have proposed new cyclic values for the bandwidth probing phase of generic BBR. The basic idea is to have the maximum bandwidth probed in the earliest cycle by sending more data. Here *BBR-n_UNIT* is a scaling factor for fractions in BBR (e.g. gains). We then introduce two variables *bbr-n_gain* and *bbr-n_drain* and assign them values of 7.5 and 4.5 respectively. This gives us the pacing gain value of 1.5 to probe for more bandwidth in the Wi-Fi 5 scenario and a value of 0.9 to drain any excess queues that have been formed. The rest of the two cycles are at a steady-state value of 1. The proposed compensating change in the drain gain is critical as we need to have the average pacing gain balanced. This is to avoid larger average pacing gains as they can cause larger queues (if the bottleneck link is full) and thus larger queuing delays, higher throughputs if the bottleneck had previously been underutilized, and potentially larger delay/loss/throughput impacts on cross-traffic. Correspondingly, lower average pacing gains cause shorter queues, lower queuing delays, lower loss rates, and potentially lower throughput if the bottleneck goes underutilized.

**Algorithm 1a: BBR-n-pacing**

**Input:** BBR-n_UNIT, bbr-n_gain, bbr-n_drain

1: **int** pacing_gain() = {

 BBR-n_UNIT * (bbr-n_gain)  / 5,                                  /* probing  for bw in Wi-Fi 5 link */

 BBR-n_UNIT * (bbr-n_drain) / 5,                       /* reducing queue for fairness with other flows */

 BBR-n_UNIT, BBR-n_UNIT, BBR-n_UNIT,                 /* cruising at 1.0*bw to  utilize pipe, */

 BBR-n_UNIT, BBR-n_UNIT, BBR-n_UNIT                     /* preventing bloating of queues */

   };

   [….]

2: bw = get_bbr_max_bw()

3. min_rtt = get_bbr_min_rtt(); /* BBR model parameters */

4: **if** bbr-n-pacing_gain > BBR-n_UNIT **then**

5: cwnd = bw * min_rtt * pacing_gain;      /* BDP × gain */

6: **end if**

Now TSQ relies on TSO, which is another sophisticated mechanism used by today’s fast NICs to relieve the CPU from the task of segmenting the data chunks and letting NICs do the work. The important point here is that the generic BBR v2 uses its mechanism of selecting a TSO frame depending on flow rates. So for a pacing rate below 1.2Mbit/sec, BBR v2 uses a min TSO/GSO burst size of 1 MSS, and above that pacing rate, it uses a TSO/GSO burst size of 2 MSS. This limit although good for a wired scenario is not suitable for Wi-Fi 5 where we need larger payloads for frame aggregation purposes. Hence, the need for *Algorithm 1b*. TSO on one end is controlling how large a burst can be and TSQ on the other end tackles how much to enqueue to make sure that queues on the sending host – including both qdisc and the NIC transmit queues can optimally feed the qdisc layer and the NIC to ensure full utilization. Now for wired networks, TSQ enqueues data that can be sent in 1 ms of time intervals for a particular flow. This limit although good for wired networks has proven [43] to be low for wireless networks Wi-Fi 5 where this hampers the frame aggregation logic of building bigger frames. We relaxed this limit via the TSQ patch to 4 ms to enqueue more packets for building bigger frames and providing a larger MSS budget for TSO *Algorithm 1b*.

The other important change is in the TSO mechanisms being used in generic BBR (BBRv2). To gradually deal with the overheads involved per packet, the Linux TCP often schedules an aggregate of TCP maximum segment size (MSS) at the sending side as a single quantum. The BBR v2 congestion control algorithm makes this decision and controls the size of this quantum that specifies the maximum size of these transmission aggregates. The decision is a trade-off as with a smaller quantum at lower data rates we face shorter bursts, less queuing delay, and a low rate of packet loss. On the other hand, a larger quantum at higher data rates such as in Wireless AC results in better throughput and lower CPU overhead.

Linux TCP uses a standard value of 2 MSS for defining how large a burst can be. This value again good for wired ethernet scenarios does not suit the Wi-Fi 5, where large data units are needed to build larger frames. Generic BBR does not follow Linux TCP here and has its own TSO bursts sizing mechanism, which we have found is also an issue behind BBR v2 not exploiting the relaxation provided in TSQ [29]. So, we propose *Algorithm 1b*. to tackle this issue. Here BBR-n-SetSendQuanta is run to update *BBR-n-send_quanta* using the *BBR-n pacing rate* and its Min/Max is computed based on the size of MSS given by *BBR-n*.*base*. For rates below 1.2 Mbps, BBR-n.base is only a single TCP MSS. For higher rates, the aggregate is made up of four TCP MSS.

**Algorithm 1b: BBR-n-TSO**

**Input**: **BBR-n-SetSendQuanta**

1: **int** BBR-n.Pacing_Rate

2: **If** (BBR-n.Pacing_Rate < 1.2 Mbps)

3: BBR-n.base = 1 * MSS

4: **Else**

5: BBR-n.base= 4 * MSS

6: BBR-n.send_quanta = **min**(BBR-n.pacing_rate * 1ms, 64KBytes)

7: BBR-n.send_quanta = **max**(BBR-n.send_quanta, BBR-n.base)

Since generic BBR is overriding the Linux TSO defaults, we had to rebuild our proposed BBR-n with the TSO modifications in the code and have it loaded in the kernel as a loadable module. The BBR-n patch, along with results from our real physical testbed for different BBR pacing gain and TSO/TSQ values are available at our online repository. The two proposed *Algorithms 1a*. *& 1b*. when deployed together with the recommendations made in the startup gain of BBRgen in (5) plays a key role in achieving increased throughput for Wireless-AC.

## 4 Methodology

**Image Source:** Physical Testbed, “Client, Wireless Router and Server icons” from Microsoft Visio Pro, technology and electronics section. https://www.microsoft.com/en/microsoft-365/visio/ CC BY 4.0.

**Fig 7:**
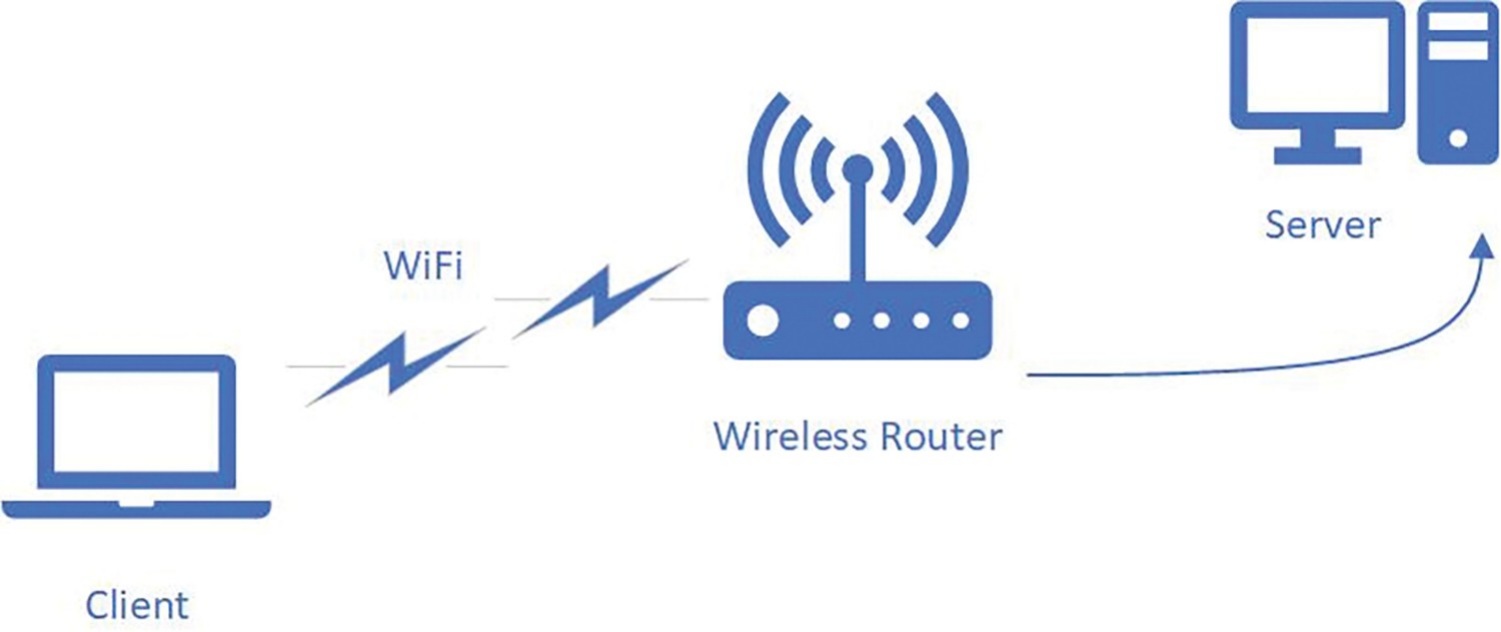
Physical testbed.

Our physical testbed is described in this section and is shown in “**Fig 7”**. It consists of a wireless client, a wireless router, and a wired server. Both client and server are running Linux Ubuntu 5.19.9 and 5.15.0-48 respectively.

Netperf 3 [44] server has been set up at the wired Linux Ubuntu server. The router from TP-Link (Archer C6) fully supports Wi-Fi 5 wireless mode. The client, which is a Linux Ubuntu machine, is using a Qualcomm QCA9377 SoC (system on chip) PCIe-based adapter using 256-QAM in a 5 Ghz band. One of the main advantages of this testbed is that it also depicts a very ubiquitous connectivity found in every home/office connection where desktop or notebook endpoints are connected via Wi-Fi 5 access point and the remaining part of the network is connected via ethernet cables. This testbed will help us evaluate the performance of our proposed algorithm for BBR-n in various bottleneck setups. Our Linux-based client will be using various TCP congestion control algorithms as given in **“Table 2”**, namely: BBRgen, BBR-n, Cubic, Reno, DCTCP, and Veno. The client will also be setting different TSQ (TCP Small Queues) values starting from the default value of 1ms and then doubling it slowly and gradually to note down its effect.

**Table 2:** Testbed specifications.

Related Parameters	Corresponding Values
Clients Kernel Version	5.13.12, 5.15.72 & 5.19.9
Servers Kernel Version	5.15.0-48 & 5.19.9
TCP congestion control	BBRgen, BBR-n, Cubic, Reno, DCTCP & Veno.
TCP Small Queues (TSQ)	TSQ (default), 2 TSQ, 4 TSQ, 8TSQ, 16 TSQ
TSO Burst sizes	1,2,4 MSS
Queueing Disciplines	FQ and FQ_Codel
Wireless chipsets	Qualcomm QCA9377
	Dlink 8812BU
	Realtek RTL8821CE
Wireless Driver	rtl88x2bu,ath10k,e1000
Tests	1/4/8/12 TCP Uploads and Real-time Response under Load Test (RRUL)
Metrics	ICMP Latency (ping RTT) TCP Throughput

All the experiments done and produced in this paper are done with the help of the Flent [45] tool, which is a flexible network tester that can run tests consisting of many bulk data flows and measure the latencies involved in the process. This makes it possible to take real-time measurements over different network topologies and collect many performance metrics. The organization of the tests performed is as follows. We begin with a single TCP upload test and compare the performance of BBR-n with BBRv2 (BBRgen) and other CCAs. Each test runs for 60 seconds, 5 initial seconds with only ICMP traffic, 40 seconds in the middle for upload traffic, and the last 5 seconds again the ICMP traffic is sent. TCP pacing rate and CWND statistics were gathered and plotted. Next is the Realtime Response Under Load (RRUL) test [46] which is a stress test. It runs a total of eight simultaneous TCP flows (four in each upload/download direction) and measures latency using both UDP and TCP packets. The parameters we used to configure the testbed of our experiments are given in **“Table 2”.**

## 5 Results and discussion

In this section results gathered from the physical testbed are shared. We will be dividing this section into four sub-sections. In these results of the experiments for **BBR-n with other CCAs**, **Pacing Rate and CWND statistics** tests for BBR-n and BBRgen, **TCP upload 1/4/8/12 streams**, and **RRUL (Realtime Response Under Load)** tests will be shared. Each experiment has been performed ten times.

### 5.1 BBR-n with other CCAs

**Fig 8:**
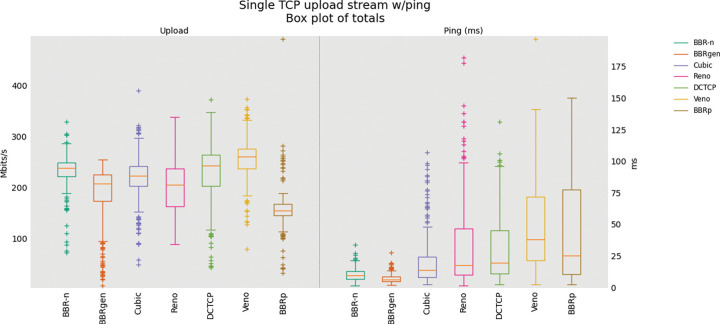
Single TCP Upload with different CCAs.

A single TCP Upload test with BBR-n and BBRgen compared with other loss-based congestion control algorithms on our physical testbed is shown in “**Fig 8”**. Analysis of variance (ANOVA) [47] was performed on the data set [14] gathered from a single TCP Upload test and the results are shown in **“Tables 3 and 4”.** The data set consists of 300 data points gathered at an interval of 0.2 seconds for 60 seconds for each of the seven CCAs. We see from **“Table 3”** that BBR-n achieved an average of 230.9 Mbps throughput as compared to 203.6 Mbps for BBRgen.

**Table 3:** ANOVA results for throughputs achieved in upload.

ANOVA: Single Factor						
SUMMARY						
*Groups*	*Count*	*Sum*	*Average*	*Variance*		
TCP upload - BBR-n	300	69270.16	230.9005	793.485		
TCP upload - BBRgen	300	61087.56	203.6252	951.9871		
TCP upload - BBRp	300	46253.41	154.178	618.5244		
TCP upload - Cubic	300	65241.51	217.4717	1428.973		
TCP upload - Reno	300	60713.98	202.3799	1925.602		
TCP upload - DCTCP	300	69032.49	230.1083	2812.226		
TCP upload - Veno	300	76668.51	255.5617	971.1647		
ANOVA						
*Source of Variation*	*SS*	*df*	*MS*	*F*	*P-value*	*F crit*
Between Groups	1831148.481	6	305191.4	224.8315	6.9E-222	2.102909
Within Groups	2841086.528	2093	1357.423			
Total	4672235.008	2099				

**Table 4:** ANOVA results for ping for CCAs.

ANOVA: Single Factor						
SUMMARY						
*Groups*	*Count*	*Sum*	*Average*	*Variance*		
Ping (ms) ICMP - BBR-n	349	3193.715	9.151045	21.29955		
Ping (ms) ICMP - BBRgen	349	2246.076	6.435749	10.58277		
Ping (ms) ICMP - BBRp	349	13347.87	38.24606	985.4194		
Ping (ms) ICMP - Cubic	349	6043.652	17.31706	303.9511		
Ping (ms) ICMP - Reno	349	9739.731	27.90754	914.4669		
Ping (ms) ICMP - DCTCP	349	9469.566	27.13343	668.845		
Ping (ms) ICMP - Veno	349	13998.43	40.11011	969.9657		
ANOVA						
*Source of Variation*	*SS*	*df*	*MS*	*F*	*P-value*	*F crit*
Between Groups	370225.1	6	61704.18	111.4791	1.6E-124	2.102302
Within Groups	1348337	2436	553.5043			
Total	1718562	2442				

In **“Table 3”** seven CCAs of **Fig 8** are analyzed via the ANOVA test. At the top of the table, we have the summary of the sums, averages, and variances of the groups being compared using 300 data points for each of the groups. At the bottom of the table, we have the ANOVA results. The “SS” column represents the sum of the squares between and within groups. “df” represents the degrees of freedom and “MS” the mean squared values. The ratio of MS for both “between groups and within groups” is known to follow the F distribution. Therefore, to get a statistical conclusion we compare this F value calculated from the data set with the F critical value (F crit) at a significance level “α” of 0.05 in the F table. Since this value of F of 224.83 is greater than the F crit value of 2.1, the results in **“Table 3”** may be interpreted as statistically significant among the means of the group at the significance level “α” of 0.05. The P-value of 6.9E-222 is well below the threshold value of 0.05 used in the ANOVA test proving that our results are statistically significant.

The ANOVA results for the analysis performed on the Ping latency data set are shown in **“Table 4**”. We see that BBR-n latency is better than pure loss-based algorithms such as TCP Cubic and Reno. The F value of 111.4 is greater than the F crit value of 2.1, along with a P-value of 1.6E-124 well below our significance level “α” of 0.05 shows that our results are statistically significant.

### 5.2 Pacing Rate and Cwnd: BBR-n vs. BBRgen

To demonstrate the effect of the pacing rate achieved via the Gain calculated in section 3.2.1 using (4) we performed a rigorous test between BBR-n and BBRgen at various pacing rate values, 3.75 * p where ‘p’ is an integer.

**Fig 9:**
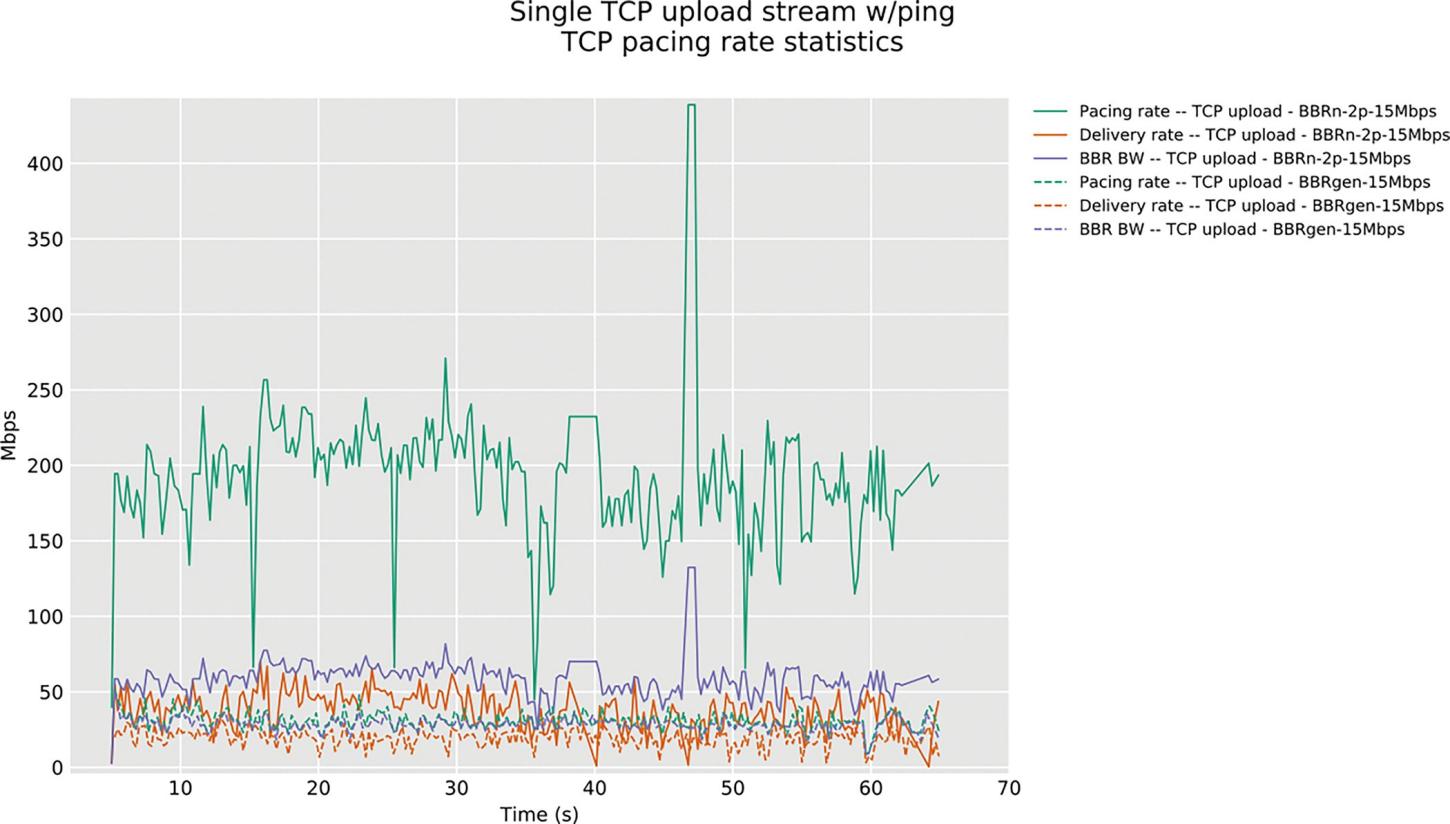
One TCP stream in upload, BBRgen vs. BBR-n pacing rate and BBR BW model.

It is evident from “**Fig 9”** below that with an increased pacing rate as proposed in our BBR-n *Algorithm 1a*. with p=2 the corresponding bandwidth and most importantly, the delivery rate is high. It is more than twice that of generic BBR.

The results from other values of ‘p’ and their effect on the pacing rate have been included in our online data source.

**Fig 10:**
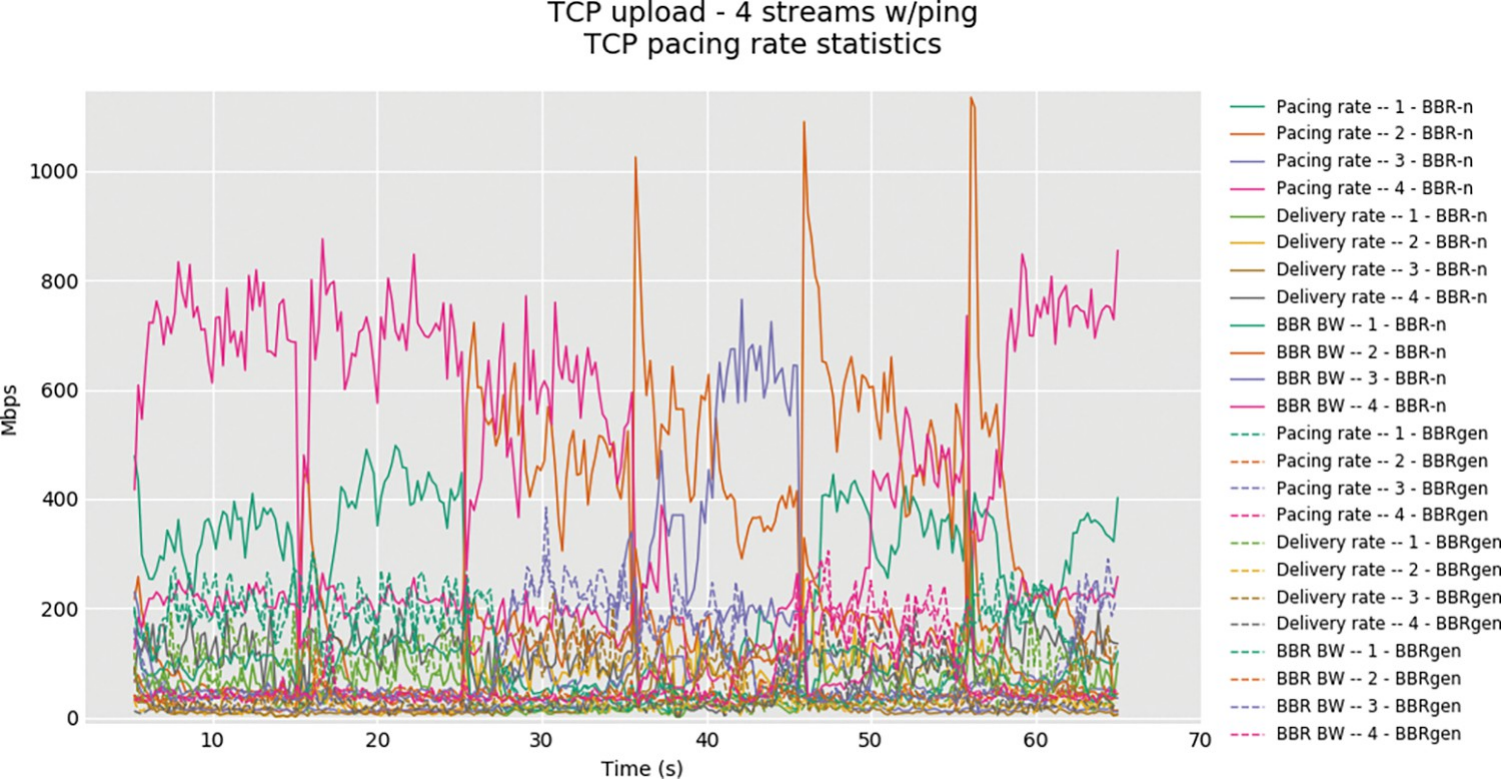
Four TCP streams in upload, BBRgen vs. BBR-n: Pacing rate and BBR BW model.

Results from the 4 TCP Upload stream tests for BBR-n and BBRgen showing the pacing rate statistics were also fetched via Flent after enabling the socket statistics of the Linux kernel. The result shown in “**Fig 10”** proves that BBR-n can pace the TCP segments much better than the generic BBR and it results in an increased delivery rate which is needed to build larger frames in Wireless-AC.

**Fig 11:**
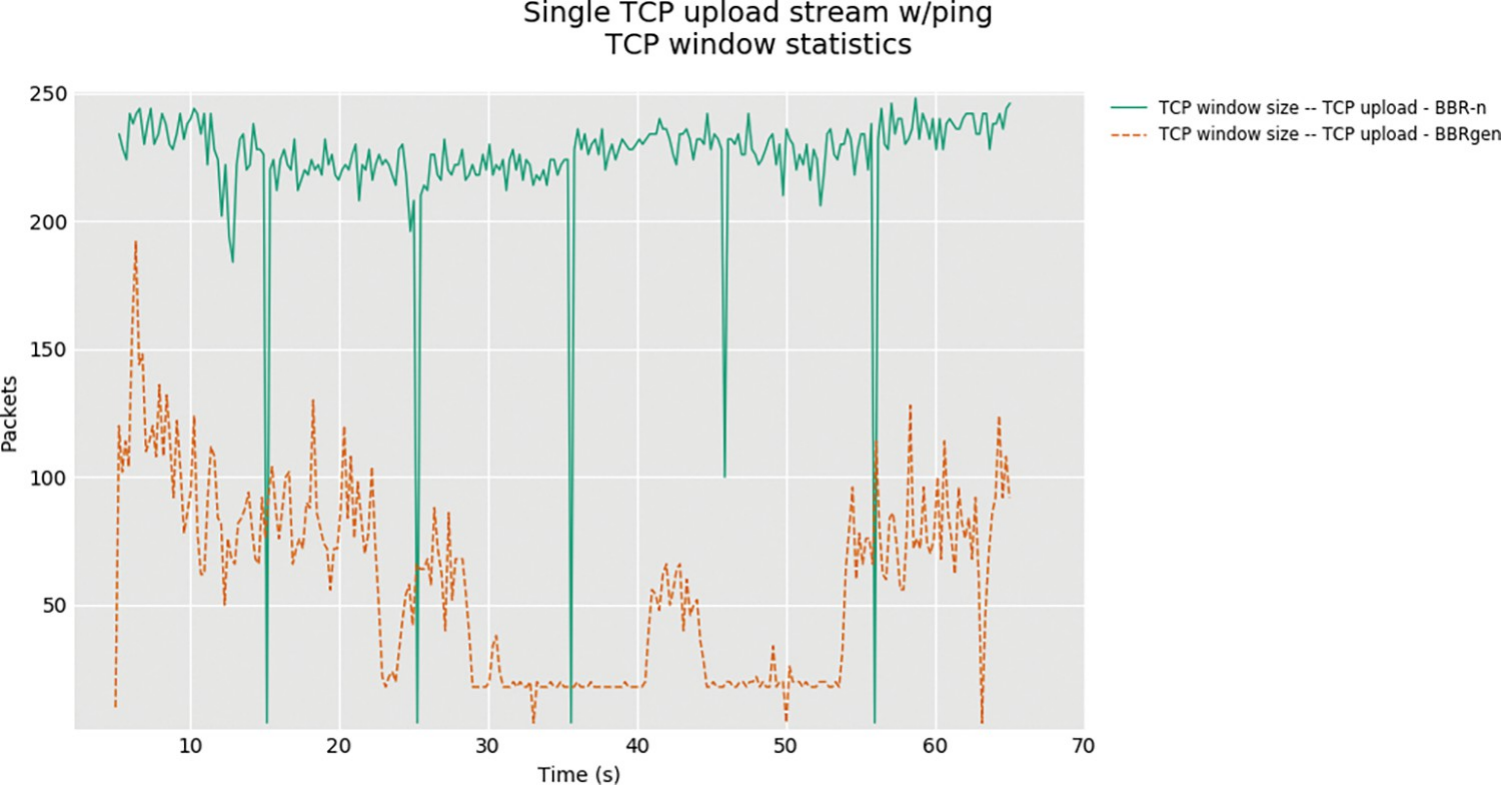
TCP Window statistics for BBR-n vs. BBRgen.

In “**Fig 11”** the resulting congestion windows were compared for both algorithms and again our proposed BBR-n with pacing rate and TSO/GSO logic streamlined proved to provide more packets than the generic BBR.

### 5.3 TCP upload test

First, we did a single TCP flow upload test via FLENT [45] to our wired Linux server which tried to consume all the available bandwidth that is presented to our Wi-Fi 5 bottleneck. In this test, we compared our proposed congestion control algorithm BBR-n with generic BBR, along with Cubic, Reno, DCTCP, and Veno. From the single stream upload test, we can deduce that although loss-based CCAs gave better throughput, the latencies involved were much higher than what we experienced in model-based CCAs such as generic BBR and our proposed BBR-n.

**Fig 12:**
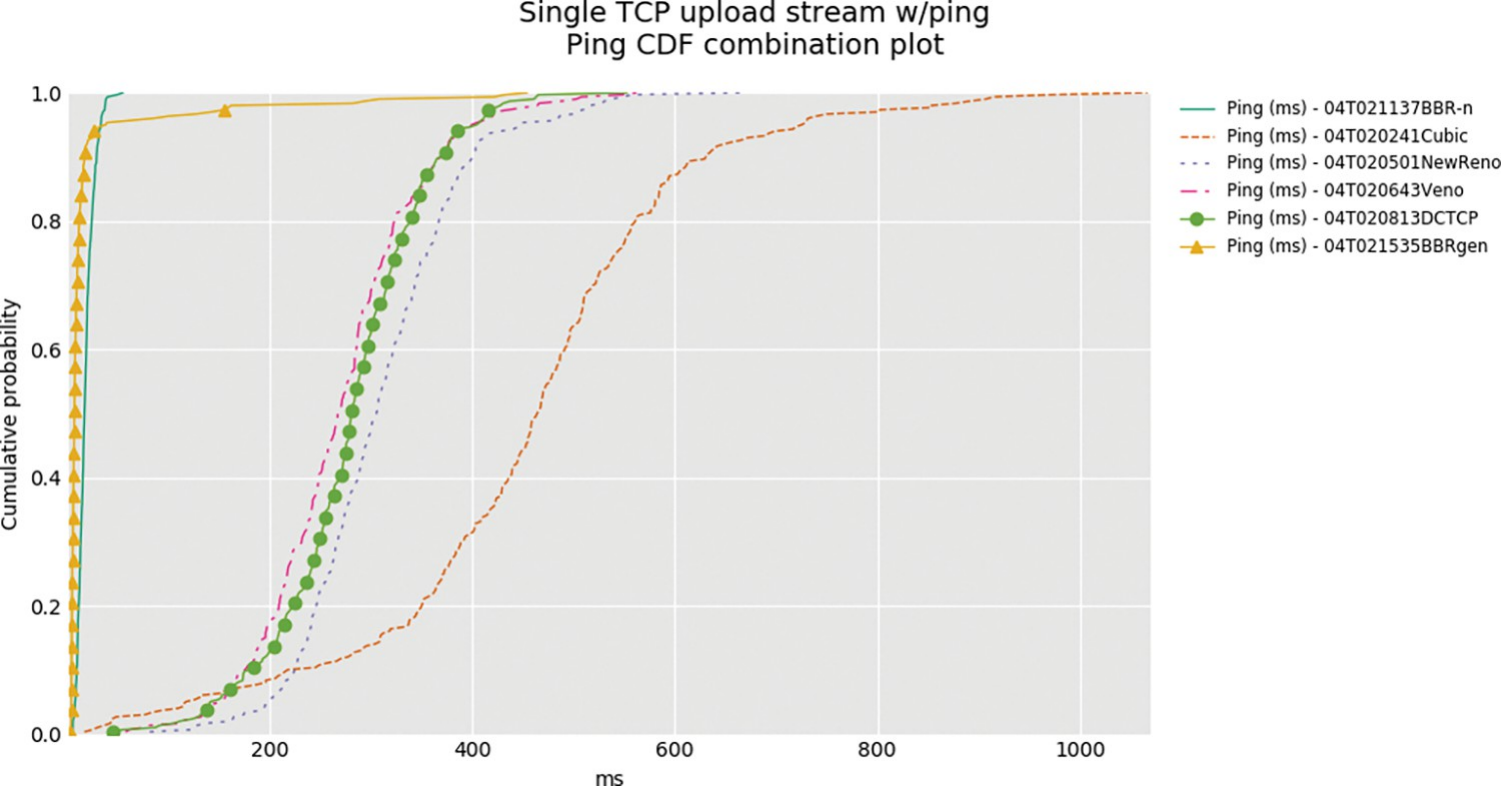
Combined Ping CDF plot for all CCAs.

The cause for this high latency is the bufferbloat phenomenon, which is evident from the box-whisker plot as shown in “**Fig 8”**. Here we see that BBR-n, with our new proposed *Algorithms 1a*. *and 1b*. is performing better than generic BBR, and ICMP ping latency is also lower. The combined Ping Cumulative Distribution Plot (CDF) of BBR-n and BBRgen with other loss-based CCAs is shown in “**Fig 12”**. We can see very clearly that BBR latency is much lower than loss-based CCAs. BBR-n here is going neck on neck with BBRgen.

**Fig 13:**
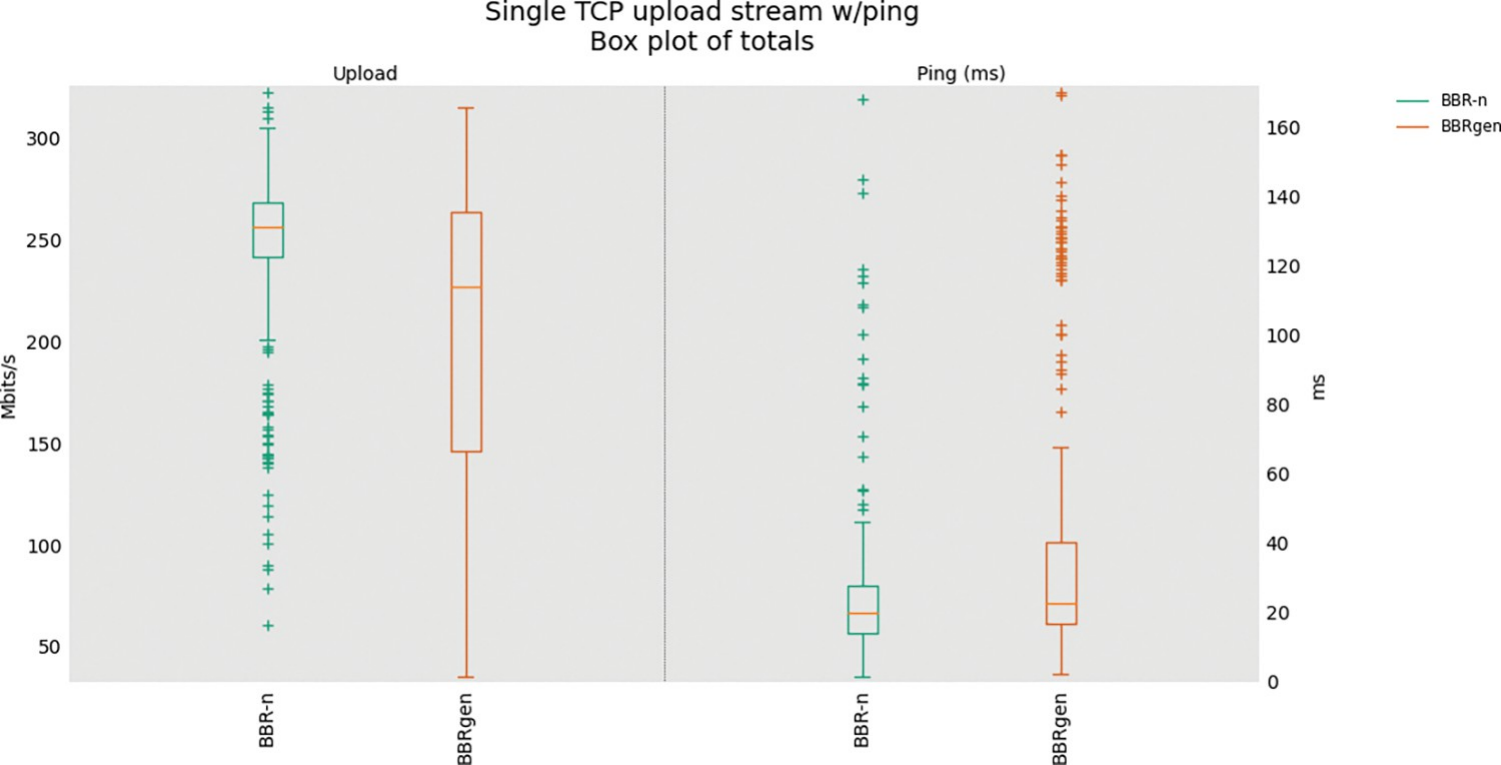
Single TCP Upload Test (BBR-n vs. BBRgen).

**Fig 14:**
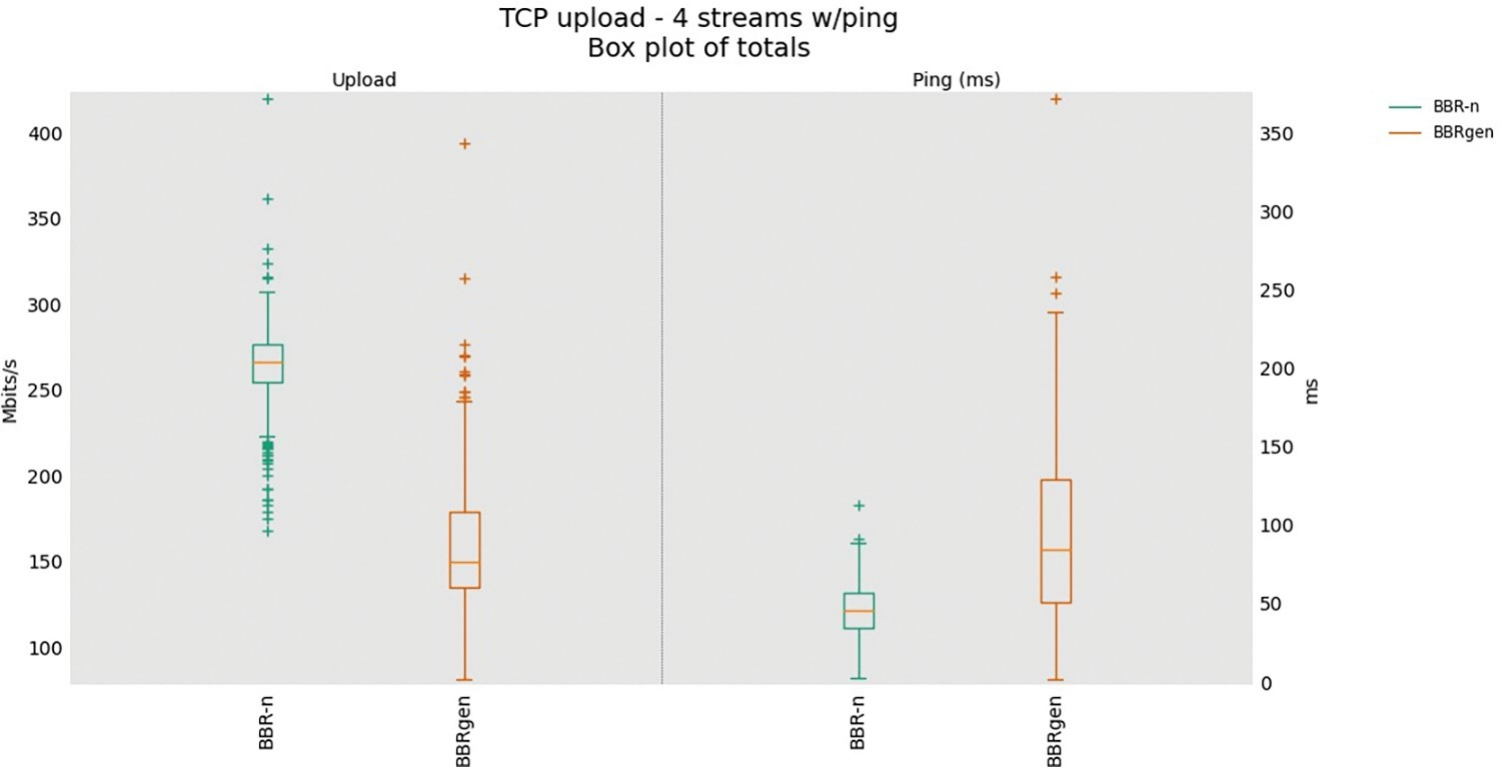
Four TCP Upload Test (BBR-n vs. BBRgen).

Now focusing on generic BBR and BBR-n, first, we did a single TCP upload test for BBRn and BBRgen and then a four upload streams test via FLENT [48] using our same physical testbed. BBR-n with our proposed *Algorithms 1a*. *& 1b*. gave better throughput (250 Mbps for BBR-n and 100 Mbps for BBRgen) as shown in **“Fig 13”**. For the four streams upload test as shown in “**Fig 14”**, BBR-n gave 260 Mbps and BBRgen gave 150 Mbps throughputs.

**Fig 15:**
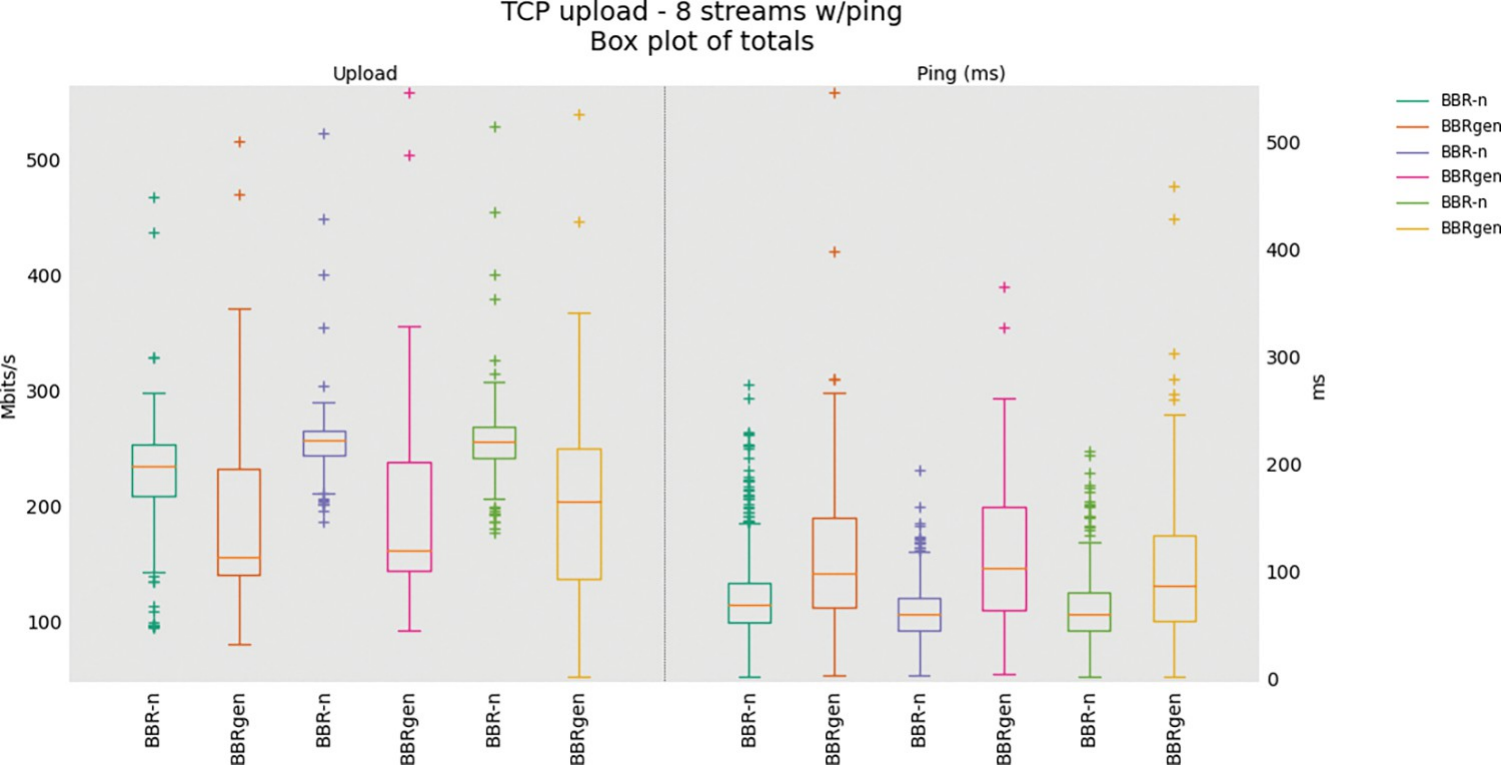
Eight TCP Uploads Test (BBR-n vs. BBRgen).

**Fig 16:**
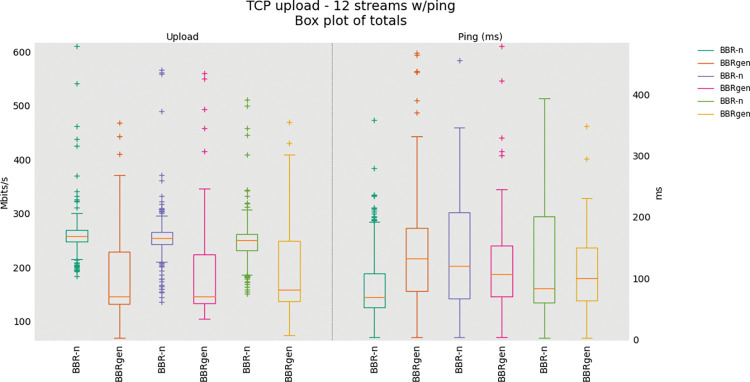
Twelve TCP Upload Test (BBR-n vs. BBRgen).

The comparison of BBR generic and BBR-n with eight/twelve TCP uploads is shown in “**Figs 15** and **16”**. Here, we have grouped multiple tests done in the same figures. It can be observed that BBR-n outperforms generic BBR and achieves higher throughput.

### 5.4 RRUL (Real Time Response Under Load) test

Next is a more rigorous test known as RRUL (Real Time Response Under Load) in which the network conditions are put into a test for upload and download. In this test, there were four TCP streams in upload and four streams in download. The TSQ and TSO budgets were kept as defaults for the Linux kernel 5.13.12 at 1 ms and 2 MSS respectively. The ICMP ping results were also computed on the way. We used this test to compare the performance of BBR-n and generic BBR and further validate that our proposed algorithm BBR-n outperforms the generic BBR in this rigorous testing scenario.

**Fig 17:**
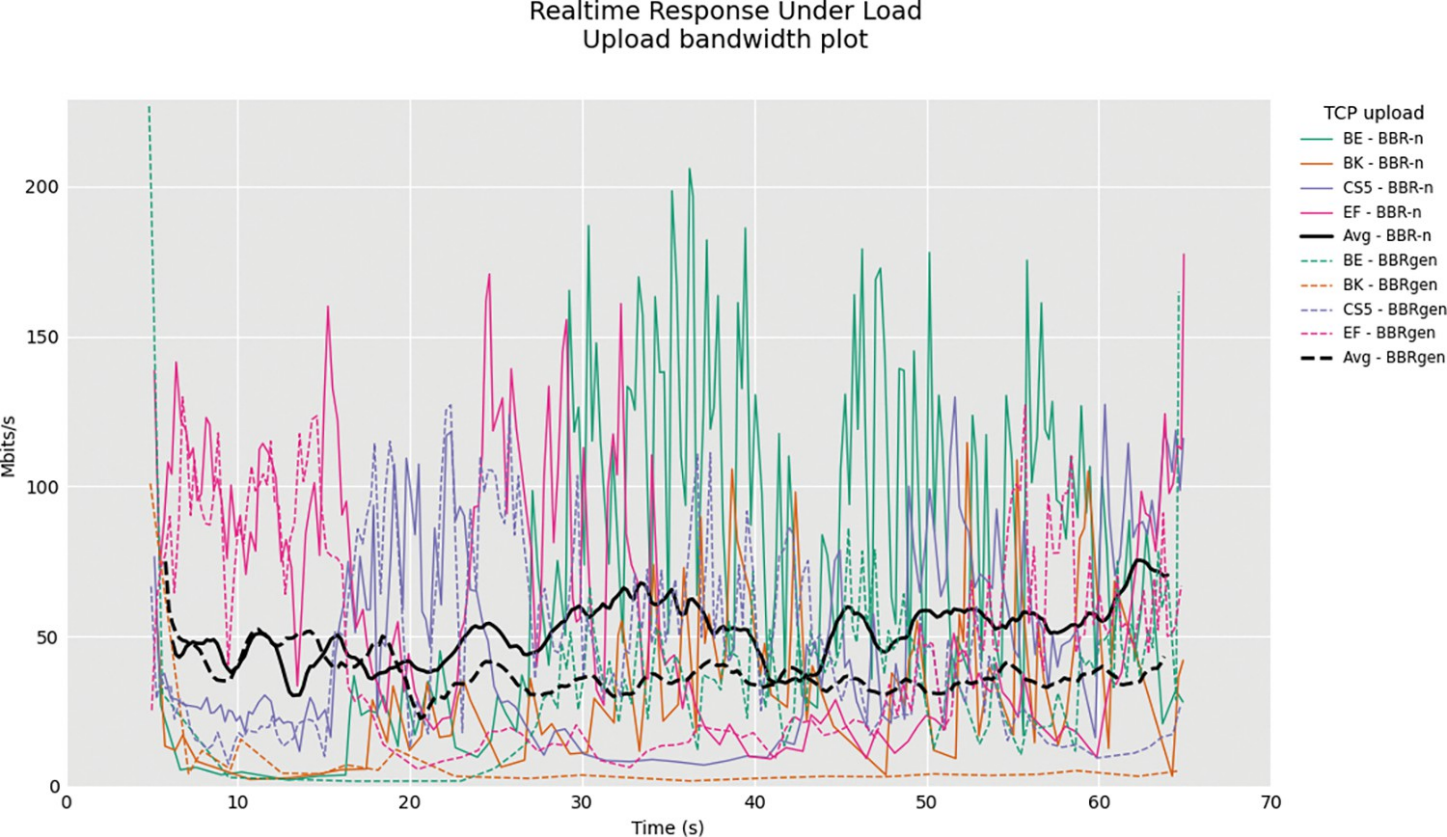
RRUL Test: For BBRgen and BBR-n Throughput (Mbps) vs. latency (ms).

**Fig 18:**
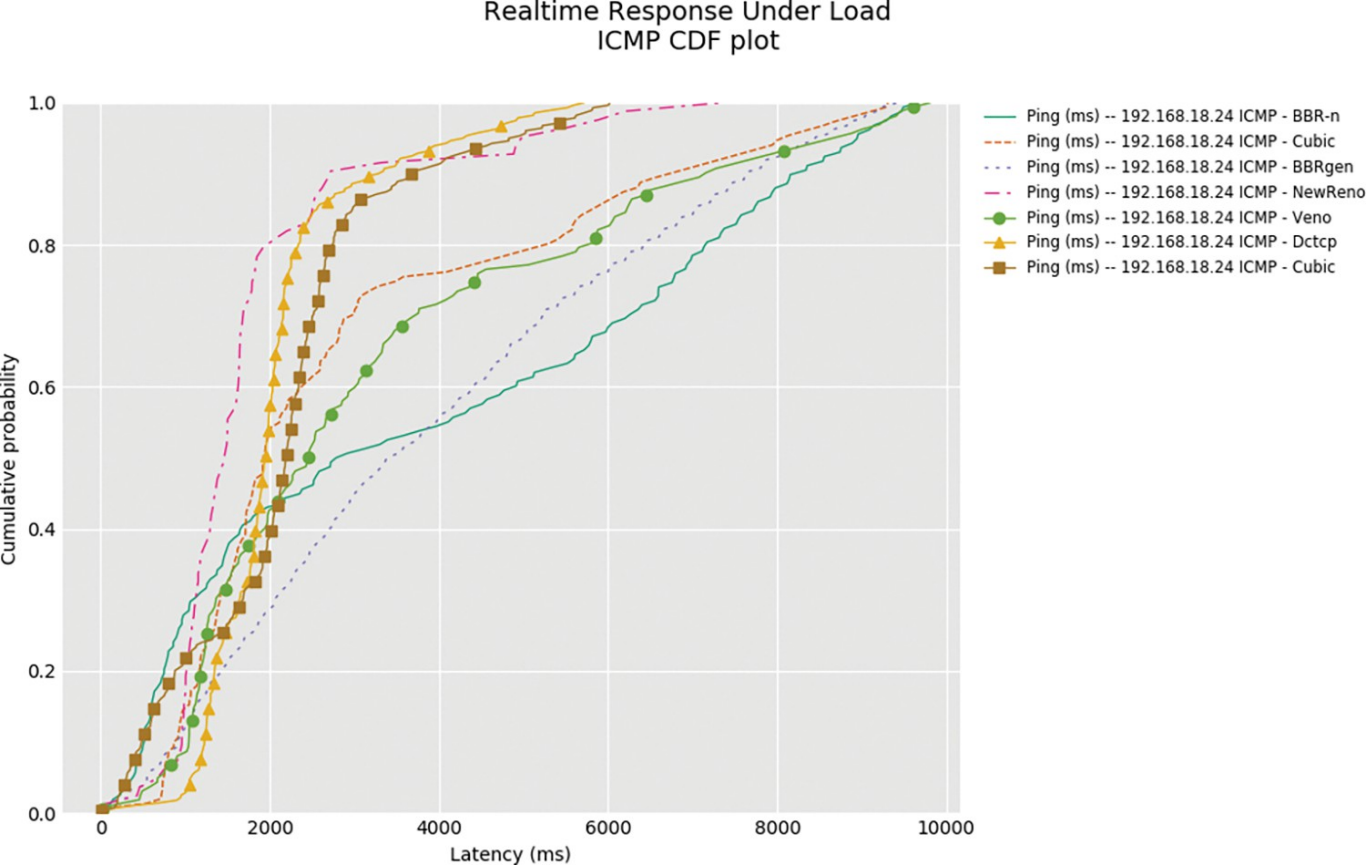
ICMP CDF Plot of BBR-n, BBRgen, and different TCP CCAs.

BBR-n outperforms BBRgen in the upload RRUL test as shown in “**Fig 17”** upload bandwidth plot. Several tests were performed, and the results are shared as a zip file at the cloud data source. The ICMP cumulative distribution plot (CDF) with other loss-based CCAs shown in “**Fig 18”** clearly shows the BBR-n latency is lower than loss-based CCAs and almost close to BBRgen in this test done in a highly congested network condition.

**Fig 19:**
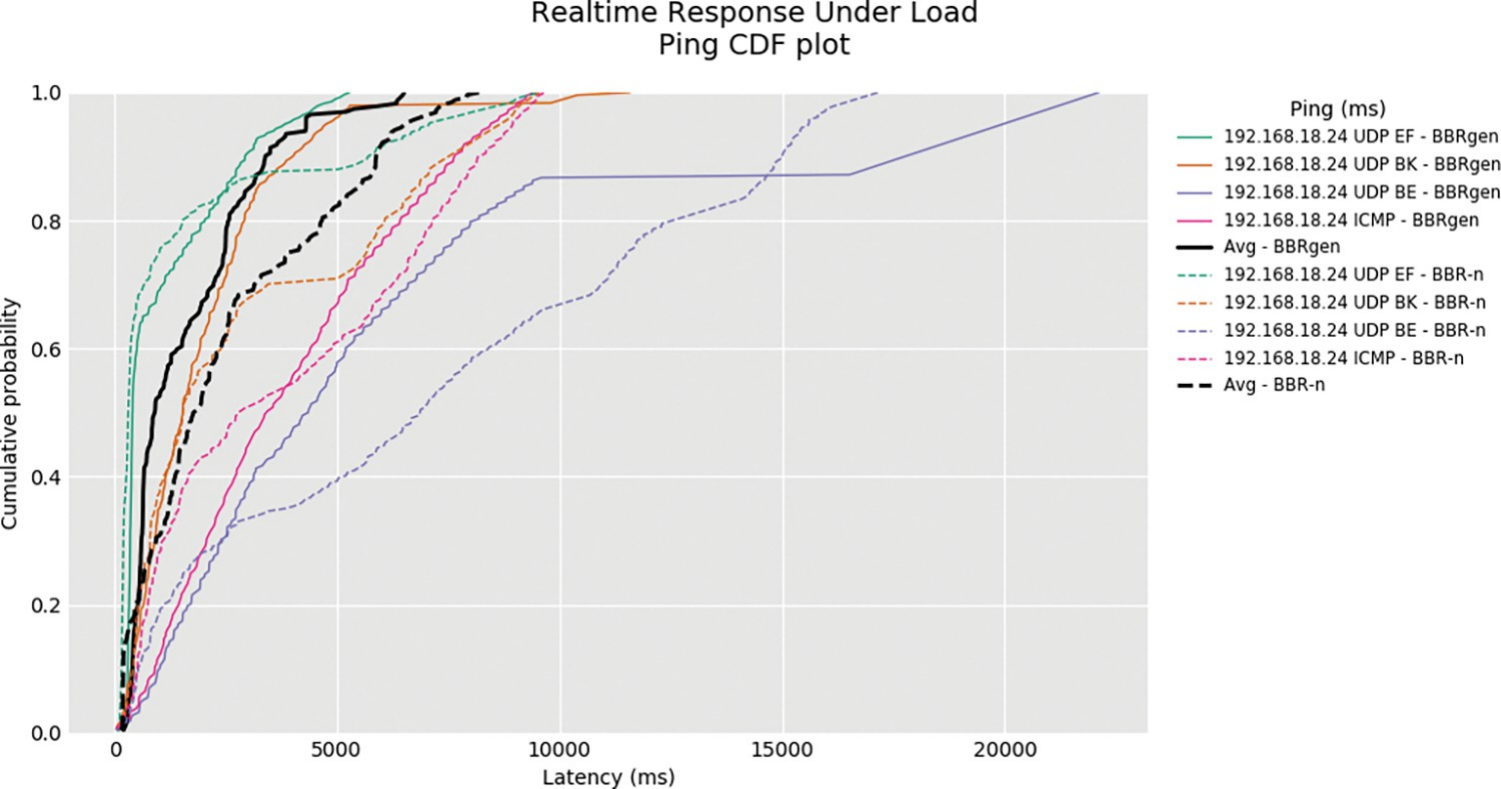
Ping CDF Plot of BBR-n, BBRgen.

**Fig 20:**
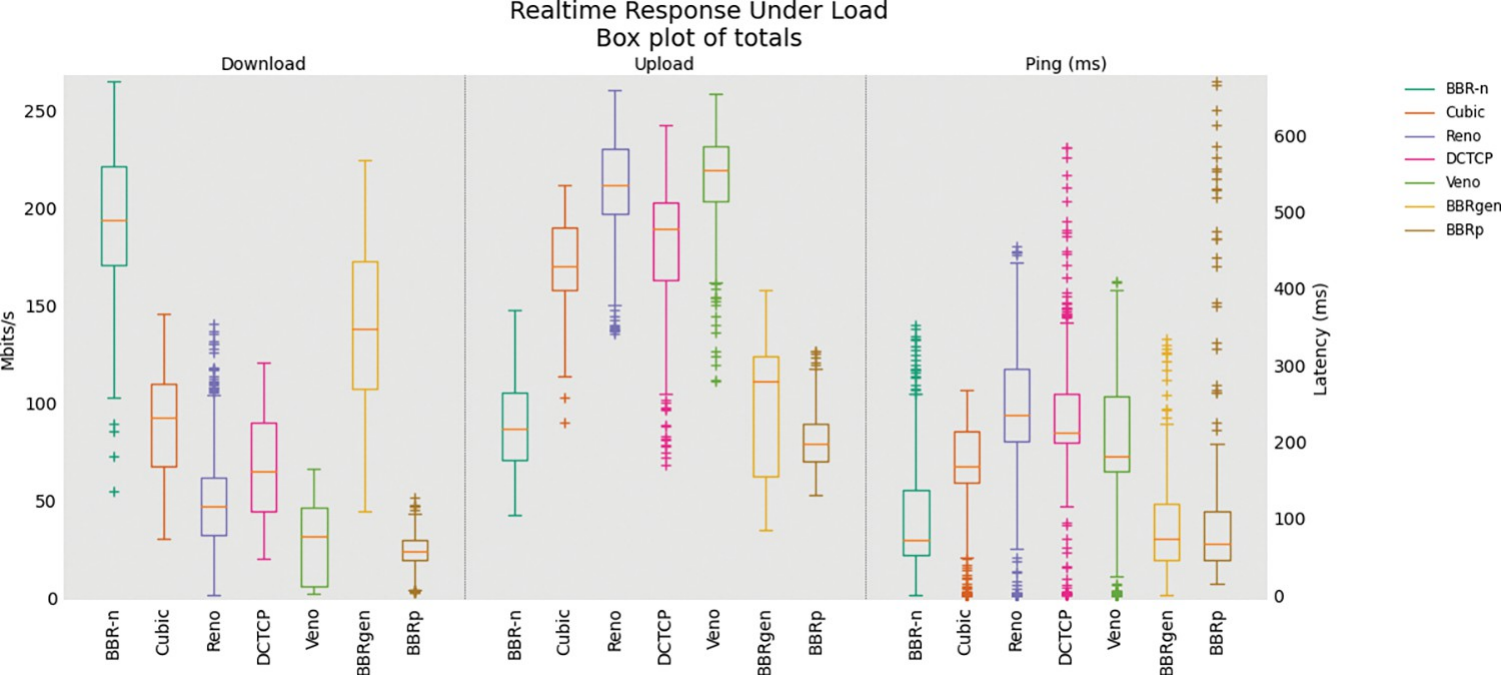
RRUL Test: BBR-n with rest of CCAs.

The Ping CDF plot was generated via the RRUL test and is shown in “**Fig 19”**. The RRUL test uses four streams in upload and four streams in download to load the network fully. It works by calculating RTT using ICMP ping and UDP round-trip measurements. This gives a true picture of the latency involved. From the output result of this test, it is clear that BBR-n average latency was less than the generic BBR. Comparing the performance with loss-based CCAs such as Cubic, Reno, Dctcp, and Veno in the RRUL test shown in “**Fig 20”**. BBR-n performance is better due to our proposed *Algorithms 1a*. and *1b*. being used in BBR-n.

The results from the ANOVA tests performed on the data set of Fig 20 are shown in **“Tables 5–7**”. From **“Table 5”** we see that BBR-n provides a throughput of 193.74 Mbps as compared with 139.86 Mbps for BBRgen. The ANOVA results show that the F value of 961.6 is larger than the critical value of 2.1. The P-value of zero indicates that the difference between the means of the groups is statistically significant (as α was 0.05). In “Table 6” BBR-n and BBRgen both are higher in throughput as compared to BBRp and pure loss-based algorithms such as TCP Cubic and Reno. The results from the ANOVA test in this case give an F value of 646.2 which is larger than the critical value of 2.1, along with a P-value of zero proving that the results are statistically significant.

**Table 5:** ANOVA results for RRUL test with all CCAs in download.

ANOVA: Single Factor						
SUMMARY						
*Groups*	*Count*	*Sum*	*Average*	*Variance*		
TCP download sum - Cubic	301	27750.22	92.19342	968.6915		
TCP download sum - Reno	300	15348.03	51.16009	1212.513		
TCP download sum - DCTCP	301	20376.33	67.69546	1075.881		
TCP download sum - Veno	300	8492.571	28.30857	601.7204		
TCP download sum - BBR-n	301	58318.08	193.7478	2033.714		
TCP download sum - BBRgen	301	42100.84	139.8699	2426.912		
TCP download sum - BBRp	302	7331.154	24.27535	165.721		
ANOVA						
*Source of Variation*	*SS*	*df*	*MS*	*F*	*P-value*	*F crit*
Between Groups	6992682	6	1165447	961.6243	0	2.102897
Within Groups	2543897	2099	1211.957			
Total	9536579	2105				

**Table 6:** ANOVA results for RRUL test with all CCAs in upload.

ANOVA: Single Factor						
SUMMARY						
*Groups*	*Count*	*Sum*	*Average*	*Variance*		
TCP upload sum - Cubic	300	52610.28	175.3676	1098.999		
TCP upload sum - Reno	300	63725.34	212.4178	2303.567		
TCP upload sum - DCTCP	300	54529.67	181.7656	2024.635		
TCP upload sum - Veno	300	65483.94	218.2798	2661.862		
TCP upload sum - BBR-n	300	28500.13	95.00941	1180.679		
TCP upload sum - BBRgen	300	29709.61	99.03856	1413.931		
TCP upload sum - BBRp	300	24563.75	81.87928	565.1086		
ANOVA						
*Source of Variation*	*SS*	*df*	*MS*	*F*	*P-value*	*F crit*
Between Groups	6258766	6	1043128	646.212	0	2.10293
Within Groups	3362419	2083	1614.219			
Total	9621185	2089				

**Table 7:** ANOVA results for ping for all CCAs in the RRUL test.

ANOVA: Single Factor						
SUMMARY						
*Groups*	*Count*	*Sum*	*Average*	*Variance*		
Ping (ms) avg - Cubic	350	55844.96	159.557	6004.7		
Ping (ms) avg - Reno	350	79358.49	226.7385	14370.02		
Ping (ms) avg - DCTCP	350	76422.42	218.3498	15827.26		
Ping (ms) avg - Veno	350	68070.69	194.4877	15035.83		
Ping (ms) avg - BBR-n	350	30193.79	86.26797	10159.89		
Ping (ms) avg - BBRgen	350	31013.89	88.61111	9286.932		
Ping (ms) avg - BBRp	350	30949.12	88.42716	18820.87		
ANOVA						
*Source of Variation*	*SS*	*df*	*MS*	*F*	*P-value*	*F crit*
Between Groups	7422953	6	1237159	96.79249	2.1E-109	2.102294
Within Groups	31199780	2441	12781.56			
Total	38622733	2447				

In **“Table 7”** ping results statistics are shown with BBR-n at 86.2 ms as compared with 88.6 ms of BBRgen and comparatively higher latencies shown by pure loss-based algorithms such as TCP Cubic and Reno.

The statistical results from **“Tables 5–7”** gathered from the ANOVA test confirm our claim that BBR-n provides better throughput than BBRgen and much lower latency when compared with pure loss-based TCP variants such as Cubic and Reno. The statistical significance of our results is also proved by the ANOVA test’s “F” and “P-values” obtained against the corresponding “F crit” and an “α” value of 0.05. Larger “F” values imply that the means of the groups are greatly different from each other as compared to the variation of individual observations in each group. A P-value of zero strongly suggests that the results are statistically significant.

## 6 Conclusion and future directions

This paper showed that standard BBR v2 has limitations when it is used with Wi-Fi 5 in a scenario in which the client is connected wirelessly to the bottleneck and the server is on a wired network. The generic BBR v2 fails to provide enough frames to the data link layer for proper Wi-Fi 5 frame aggregation and consequently throughput is reduced. We introduced BBR-n which solved this issue by fine-tuning the startup phase of a typical BBR v2 flow by providing a revised BBR v2 startup gain. This new startup gain ensures that BBR v2 flow ramps up with a smoothly increasing pacing rate that will double each RTT. In the steady state phase, our proposed BBR-n allowed the congestion control to aggregate the packets and unleash the bottleneck bandwidth optimally. Our experiments demonstrated that BBR-n performs better than generic BBR v2 (BBRgen) in all TCP upload tests done with 1/4/8/12 streams. Also, in the RRUL test done in a highly congested environment, BBR-n performed better with increased throughput in upload as compared to BBR v2. We proved via our tests that BBR-n provides better throughput than generic BBR. More than twice the throughput was achieved for Wireless-AC while keeping fairness between the uplink and downlink paths as evident from TCP Upload and RRUL tests. BBR-n also reduces the ICMP latency to lower values than Cubic, Reno, DCTCP, and Veno. We believe that our work done in this paper on improving throughput for BBR v2 with Wi-Fi 5 will pave the way for future improvements in BBR v2 when used with Wi-Fi 6.
